# A Peptide of Heparin Cofactor II Inhibits Endotoxin-Mediated Shock and Invasive *Pseudomonas aeruginosa* Infection

**DOI:** 10.1371/journal.pone.0102577

**Published:** 2014-07-21

**Authors:** Martina Kalle, Praveen Papareddy, Gopinath Kasetty, Mariena J. A. van der Plas, Matthias Mörgelin, Martin Malmsten, Artur Schmidtchen

**Affiliations:** 1 Division of Dermatology and Venereology, Department of Clinical Sciences, Lund University, Biomedical Center, Lund, Sweden; 2 Division of Infection Medicine, Department of Clinical Sciences, Lund University, Biomedical Center, Lund, Sweden; 3 Department of Pharmacy, Uppsala University, Uppsala, Sweden; 4 Lee Kong Chian School of Medicine, Nanyang Technological University, Singapore, Singapore; Fundação Oswaldo Cruz, Brazil

## Abstract

Sepsis and septic shock remain important medical problems with high mortality rates. Today's treatment is based mainly on using antibiotics to target the bacteria, without addressing the systemic inflammatory response, which is a major contributor to mortality in sepsis. Therefore, novel treatment options are urgently needed to counteract these complex sepsis pathologies. Heparin cofactor II (HCII) has recently been shown to be protective against Gram-negative infections. The antimicrobial effects were mapped to helices A and D of the molecule. Here we show that KYE28, a 28 amino acid long peptide representing helix D of HCII, is antimicrobial against the Gram-negative bacteria *Escherichia coli* and *Pseudomonas aeruginosa*, the Gram-positive *Bacillus subtilis* and *Staphylococcus aureus*, as well as the fungus *Candida albicans*. Moreover, KYE28 binds to LPS and thereby reduces LPS-induced pro-inflammatory responses by decreasing NF-κB/AP-1 activation *in vitro.* In mouse models of LPS-induced shock, KYE28 significantly enhanced survival by dampening the pro-inflammatory cytokine response. Finally, in an invasive *Pseudomonas* infection model, the peptide inhibited bacterial growth and reduced the pro-inflammatory response, which lead to a significant reduction of mortality. In summary, the peptide KYE28, by simultaneously targeting bacteria and LPS-induced pro-inflammatory responses represents a novel therapeutic candidate for invasive infections.

## Introduction

Bacterial infections remain a major health care issue worldwide, not only due to increasing antibiotic resistance of various bacterial strains [1], [2], but also because of the devastating consequences of severe infections involving localized organ damage or systemic inflammatory responses, e.g., as seen in sepsis [3], [4]. The latter significantly contributes to the high mortality rates recorded for sepsis and septic shock, reaching up to 50% [3], [5]. Despite antibiotic usage and improved intensive care the incidence of sepsis has increased during in the last two decades, and now reaches about 240 per 100000 people in the US [6]. This illustrates the importance of developing new treatment concepts, which eliminate not only the bacteria, but also control the immune system in order to prevent its detrimental over-activation during sepsis. In a recent study investigating the prevalence and outcome of infections in intensive care units worldwide, 62% of the isolated bacterial strains were Gram-negative, predominantly *Pseudomonas* species (20%) and *Escherichia coli* (16%) [4]. In this perspective, our recent discovery of a novel host defense function of the coagulation protein heparin cofactor II (HCII) in Gram-negative infections is of particular interest [7]. The molecule was found to exert a direct antimicrobial effect against *P. aeruginosa* as well as *E. coli*. The importance of HCII for clearing such infection was evidenced by decreased HCII levels observed in wild-type animals challenged with bacteria or endotoxin, as well as in HCII knock-out mice, which showed impaired bacterial clearance upon infection.

Coagulation and inflammation are tightly cross-linked mechanisms that play an important role during infections, especially sepsis [8]. Thus, using regulatory coagulation proteins for infection treatment is a possible therapeutic approach, previously explored in infection models and clinical trials employing various modulatory proteins, such as the serpin antithrombin III [9], recombinant tissue factor pathway inhibitor 1 [10]–[12] or recombinant activated protein C [13]. Unfortunately, none of the above was successful [9], [13], [14], indicating a need for new anti-infective drugs.

Antimicrobial peptides (AMP) have recently attracted much interest as novel anti-infective drug candidates [15]–[17]. Multifunctional AMPs, also termed host defense peptides play an essential role in the innate immune system for fighting invading pathogens [15], [18]. They are small structures (≈10–30 aa), mostly cationic, hydrophobic molecules with direct and rapid antimicrobial action against a broad-spectrum of both Gram-negative and Gram-positive bacteria, as well as fungi [17], [19]–[21]. In addition to their antimicrobial properties, many of these peptides have also been found to exert various other functions including immunomodulation of pro- or anti-inflammatory pathways, dendritic cell activation, and stimulation of wound healing and angiogenesis [22]–[24].

Previous studies on HCII revealed that its antimicrobial function was mediated by the two heparin-binding regions helix A and D [7], [25]. The 28-mer peptide, KYE28 of helix D is cationic and amphipathic [26], features shared with not only a helix A-derived peptide of HCII, but also the classical cathelicidin LL-37 [27] as well as C-terminal peptides of human thrombin, previously shown to be effective in experimental models of endotoxin shock and/or invasive infection [24], [25], [28], [29].

With this information as background, we here set out to investigate whether KYE28 could act as functional mimic of the host defense actions of HCII. We explored its antimicrobial and immunomodulatory effects *in vitro*, as well as in mouse models of endotoxin shock and invasive *P. aeruginosa* infection.

KYE28 displayed a broad antimicrobial spectrum. Moreover, the peptide significantly reduced endotoxin as well as bacterial-induced pro-inflammatory cytokine responses. This combination of antimicrobial and anti-inflammatory effects significantly increased survival in experimental models of endotoxin shock and invasive infection with *Pseudomonas aeruginosa*.

## Materials and Methods

### Ethics statement

The use of human blood was approved by the Ethics Committee at Lund University, Lund, Sweden (Permit Number: 657-2008). Written informed consent was obtained from the donors. The animal experiments were conducted according to national guidelines (Swedish Animal Welfare Act SFS 1988∶534) and were approved by the Laboratory Animal Ethics Committee of Malmö/Lund, Sweden (Permit numbers: M75-10, M228-10, M227-10). Animals were housed under standard conditions of light and temperature, and had free access to standard laboratory chow and water.

### Peptides

KYE28 (NH_2_-KYEITTIHNLFRKLTHRLFRRNFGYTLR-COOH) and LKG23 (LKGETHEQVHSILHFKDFVNASS) were synthesized by Biopeptide Co., San Diego, USA, and LL-37 (LLGDFFRKSKEKIGKEFKRIVQRIKDFLRNLVPRTES) was obtained from Innovagen AB. The purity (>95%) of these peptides was confirmed by mass spectral analysis (MALDI-ToF Voyager).

### Microorganisms

Bacterial isolates *Escherichia coli* ATCC 25922, *Pseudomonas aeruginosa* ATCC 27853, *Staphylococcus aureus* ATCC 29213, *Bacillus subtilis* ATCC 6633, *Streptococcus pyogenes* AP1, *Streptococcus pneumonia* TIGR4, as well as *Candida albicans* ATCC 90028 and *Candida parapsilosis* ATCC 90018 were purchased from the American Type Culture Collection (ATCC). Clinical isolates of *Escherichia coli*, *Pseudomonas aeruginosa* and *Staphylococcus aureus* were obtained from the Department of Bacteriology, Lund University Hospital.

### Viable-count analysis

*E. coli* ATCC 25922, *P. aeruginosa* 15159, and *S. aureus* ATCC 29213 bacteria were grown to mid-logarithmic phase in Todd-Hewitt (TH) broth (Becton, Dickinson) and washed in 10 mM Tris, pH 7.4 containing 5 mM glucose. Following this, bacteria were diluted in 10 mM Tris, 0.15 M NaCl, with or without 20% human citrate-plasma (50 µL; 2×10^6^ cfu/mL) and incubated with KYE28 or LL-37 at the indicated concentrations, at 37°C for 10 up to 120 min. Serial dilutions of the incubation mixtures were plated on TH agar, followed by overnight incubation at 37°C and subsequent determination of the number of colony forming units (cfu). Hundred percent survival was defined as total survival of bacteria in the same buffer and under the same condition in the absence of peptide.

### Radial diffusion assay

The radial diffusion assay **(**RDA) was performed as described previously [30], [31]. In brief, bacteria were grown to mid-logarithmic phase in 3% w/v trypticase soy broth (TSB; Becton, Dickinson). After a wash and dilution in 10 mM Tris, pH 7.4, 4×10^6^ cfu were added to 15 mL of an underlay agarose gel, which was subsequently poured into a petri dish. After solidification, wells of 4 mm diameter were punched, solutions containing KYE28 or LL-37 (100 µM) were added to the wells and plates were incubated at 37°C for 3 h to allow peptide diffusion. Next, the underlay gel was covered with 15 mL of overlay gel and incubated overnight at 37°C. Antimicrobial activity of the peptides was determined by measuring the clearing zone around each well.

### Minimal inhibitory concentration determination

To determine the minimal inhibitory concentration **(**MIC) of KYE28, a microtiter broth dilution method was utilized, as previously described in the NCSLA guidelines [32]. In brief, overnight cultures of indicated bacteria were suspended and further diluted in Mueller-Hinton (MH) broth (Becton, Dickinson). KYE28 was dissolved in H_2_O to concentrations ranging from 320 µM to 2.5 µM by serial dilution. Fifty µL of each concentration was added to the corresponding well of a 96-well microtiter plate (polypropylene, Costar Corp.) together with 50 µL of bacteria (2×10^5^) in MH broth. The plate was incubated at 37 °C for 16–18 h. The MIC was obtained as the lowest concentration where no visual growth of bacteria was detected.

### Scanning electron microscopy

In order to visualize effects of KYE28 on *P. aeruginosa* 15159 and *S. aureus* ATCC 29213, bacteria were grown to mid-logarithmic phase in TH broth, washed, diluted (1–2×10^6^ cfu/sample) and incubated for 2 h at 37°C in the presence or absence of 30 µM KYE28. Subsequently, bacteria were fixed in 2.5% glutaraldehyde in 0.15 M sodium cacodylate buffer, pH 7.4, overnight at room temperature and prepared as described previously [24]. For sample examination a Philips/FEI CM 100 electron microscope operated at 80 kV accelerating voltage was used, and images were recorded with a side-mounted Olympus Veleta camera at the Core Facility for integrated Microscopy (CFIM), Copenhagen University, Denmark. Further, scanning electron microscopy was performed on lungs from mice 20 h after LPS injection or 12 h post-challenge with *P. aeruginosa*. Samples were prepared as described previously [24] and examined with a JEOL JSM-350 scanning electron microscope. For the quantification of pulmonary lesions, lung samples from 30 different fields covering an entire lung sections were prepared, and the percentage of fibrin deposits and fields exhibiting haemorrhage was assessed [24].

### LPS models *in vitro*

RAW 264.7 mouse macrophages (3.5×10^5^cells/well) (ATCC) were seeded in 96-well tissue culture plates (Nunc) in phenol red-free Dulbecco's modified Eagle medium (DMEM; PAA laboratories), supplemented with 10% (v/v) heat-inactivated fetal bovine serum (FBS; Invitrogen) and 1% (v/v) Antibiotic-Antimycotic solution (ASS; Invitrogen). Cells were stimulated with 10 ng/mL *E. coli* LPS (0111:B4) (Sigma-Aldrich, approximate 500.000 endotoxin units/mg) with and without KYE28 in the indicated settings. Human blood, anticoagulated with lepirudin was diluted (1∶4) with RPMI 1640, glutamax (Gibco) and stimulated with 100 ng/mL *E. coli* LPS (0111:B4), again with or without the indicated concentrations of KYE28 for 20 h, before supernatants were collected for cytokine analysis.

### NF-κB activation assay

The NF-κB reporter cell lines RAW-Blue and THP1-XBlue-CD14 purchased from InvivoGen were cultured according to manufacturer's instructions. THP1-XBlue-CD14 or RAW-Blue cells were stimulated with 100 or 10 ng/mL *E. coli* LPS (0111:B4), respectively, together with the indicated concentrations of KYE28 for 20–24 h. THP1-XBlue-CD14 cells were also stimulated with 1 µg/mL lipoteichoic acid (LTA, InvivoGen), 10 µg/mL zymosan (InvivoGen), 1 µg/mL *E. coli-*derived peptidoglycan (PGN-EB, InvivoGen), 20 ng/mL PAM_3_CSK_4_ (InvivoGen) or 100 ng/mL phorbol 12-myristate 13-acetate (PMA, InvivoGen) (see Figure S1) with or without addition of KYE28 or the control peptide LKG23. In other experiments RAW-Blue cells were treated with either KYE28 or LPS for 1 h, before addition of LPS (pretreatment) or KYE28 (post-treatment). Measurement of NF-κB/AP-1 activation was done using the Quanti Blue assay according to the manufacturer's protocol. Briefly, upon stimulation both cell lines produce secreted embryonic alkaline phosphatase (SEAP), which was detected in cell supernatants by using a SEAP detection reagent and analysis of the absorbance at 600 nm.

### LPS model *in vivo*

Male C57BL/6 mice (8–10 weeks), were injected intraperitoneally (i.p.) with 18 mg/kg *E. coli* 0111:B4 LPS or 36 mg/kg *P. aeruginosa* LPS (serotype 10) (Sigma-Aldrich). Thirty minutes after LPS injection, 250 µL of KYE28 (0.5 mg/mouse) or buffer alone were injected i.p. into the mice. Status and weight were regularly monitored for seven days. Mice showing the defined and approved endpoint criteria were sacrificed by an overdose of isoflurane (Abott) and counted as non-survivors. For blood collection and histochemistry of the lungs, mice were sacrificed 8 and 20 h after LPS challenge, and lungs were removed and fixed. In another set of experiments male and female C57BL/6 mice (8–9 weeks) were challenged with 12 mg/kg *E. coli* 0111:B4 LPS (i.p.) and treated 30 min later with 50–500 µg of KYE28. Mice were sacrificed 20 h after LPS injection and cytokine release was analyzed in the blood samples. To determine the number of platelets in the blood samples at indicated time points, the VetScan HM5 System (TRIOLAB) was utilized.

### Cytokine assa*y*

The levels of IL-6, IL-10, MCP-1, IFN-γ, and TNF-α were measured either in cell culture supernatants from RAW 264.7 cells or murine plasma using the Mouse Inflammation Kit (Becton, Dickinson) according to the manufacturer's instructions. The cytokine levels in human plasma were assessed using BioSource CytoSets (Invitrogen).

### *P. aeruginosa* infection model

*P. aeruginosa* 15159 bacteria were grown to logarithmic phase (OD_620_∼0.5), harvested, washed in PBS, diluted in the same buffer to 2×10^9^ cfu/mL, and kept on ice until injection. Hundred microliter of the bacterial suspension was injected i.p. into male C57BL/6 mice. One h or 1 and 7 h afterwards, 0.5 mg KYE28 or buffer alone was injected subcutaneously (s.c.) to the mice. In order to study bacterial dissemination to target organs spleen, liver and kidney were harvested, placed on ice, homogenized, and colony-forming units (cfu) were determined. In another experiment the animal status was regularly monitored for seven days. Mice showing the defined and approved endpoint criteria were sacrificed by an overdose of isoflurane and counted as non-survivors.

### Histochemistry

Organs collected 20 h after LPS injection were immediately fixed in 4% paraformaldehyde before they were embedded in paraffin and sectioned. Sections were stained 10 min with Mayers Hematoxilin (Histolab AB) and 7 min with Eosin (Merck).

### Statistics

Values are shown as mean with SEM. For statistical evaluation of two experimental groups the Mann-Whitney U-test was used and for comparison of survival curves the log-rank test with *p-<0.05, **<0.01 and ***p<0.001. Multiple groups were compared using either One-Way ANOVA with the Dennett's post-test or the Kruska-Wallis test with Dunn's post-test. Samples were compared to controls.

## Results

### KYE28 displays broad antimicrobial activity

**Figure 1:**
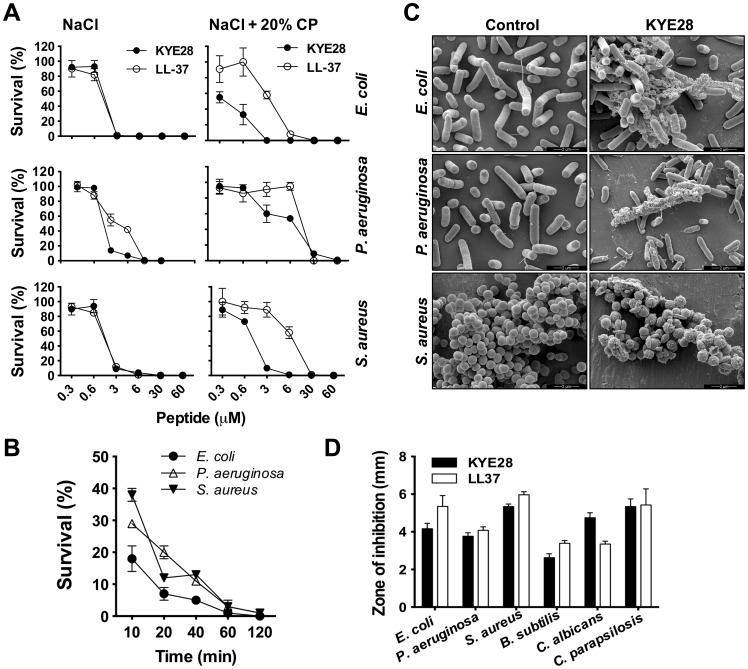
Antimicrobial effects of KYE28. (**A**) Antibacterial effects of KYE28 and LL-37 against *E. coli* ATCC 25922, *P. aeruginosa* ATCC 27853, and *S. aureus* ATCC 29213 in viable count assays. 2×10^6^ cfu/mL of bacteria were incubated with peptide in 0.15 mM NaCl, 10 mM Tris, pH 7.4 with or without human citrate plasma (CP) (n = 3). (**B**) The kinetics of bacterial killing by KYE28 (at 6 µM) in 0.15 M NaCl, 10 mM Tris, pH 7.4 containing 20% human citrate plasma was analyzed by viable count assays using 2×10^6^ cfu/mL of. *E. coli* ATCC 25922, *P. aeruginosa* ATCC 27853 and *S. aureus* ATCC 29213 (n = 3). (**C**) Effects of KYE28 on bacterial membranes were visualized by scanning electron microscopy**.** *E. coli* ATCC 25922, *P. aeruginosa* ATCC 27853 and *S. aureus* ATCC 29213 were incubated together with KYE28 (30 µM) (Control  =  buffer control)**.** **(D)** Evaluation of antimicrobial activity (using RDA) of KYE28 and LL-37 (100 µM) against the indicated microbes. The clearance zones correspond to the inhibitory effect of each peptide after incubation at 37°C for 18-24 h (n = 3).

As a first step, antimicrobial assays were performed in order to elucidate the antimicrobial spectrum width of KYE28. Results of viable count assays confirmed previous results on *E. coli* [7], but also revealed that the HCII-derived peptide displayed a significant and dose-dependent antibacterial activity against *P. aeruginosa* and *S. aureus* in Tris buffer containing physiological salt (0.15 M NaCl), as well as in presence of 20% human citrate plasma (**Figure 1A**). The human cathelicidin-derived peptide LL-37 was used as a control and displayed a similar activity. Further, kinetic studies demonstrated that 80–90% of the bacterial killing, evaluated in the presence of human plasma, occurred within 20 min (**Figure 1B** **),** indicating a fast direct action compatible with other antimicrobial peptides [31], [33], [34]. Studies employing electron microscopy in combination with previous data on liposomes [26] showed that KYE28 exerts its antibacterial effects by disrupting the bacterial cell wall which leads to leakage and cell death (**Figure 1C**). Finally, results of a screening of antibacterial effects of KYE28 were compared to those of LL-37 against several ATCC strains and clinical isolates using MIC assays (**Table 1** **)** and RDA (**Figure 1D**). The data show that KYE28 exerts significant antimicrobial effects *in vitro,* exhibiting MIC-levels compatible with previous results on LL-37 and other broad-spectrum amphipathic peptides [35], [36].

**Table 1:** MIC values of KYE28 and LL-37 for the indicated bacteria.

		MIC KYE28		MIC LL-37	
Bacterial strains		µM	mg/L	µM	mg/L
***E. coli***	ATCC 25922	10	36	20	90
	Clinical isolate 37.4	5	18	5	22
	Clinical isolate 47.1	2.5	9	5	22
	Clinical isolate 49.1	40	144	10	45
***P. aeruginosa***	ATCC 27853	10	36	10	45
	Clinical isolate 15159	20	72	20	90
	Clinical isolate 10.5	10	36	10	45
	Clinical isolate 51.1	20	72	40	180
	Clinical isolate 62.1	10	36	20	90
	Clinical isolate 18488	10	36	20	90
***S. aureus***	ATCC 29213	5	18	40	180
	Clinical isolate 16065	2.5	9	10	45
	Clinical isolate 13430	5	18	20	90
	Clinical isolate 14312	5	18	10	45
	Clinical isolate 18800	5	18	5	22
	Clinical isolate 18319	5	18	10	45
***S. pyogenes***	AP1	10	36	1.2	5
***S. pneumoniae***	TIGR4	5	18	10	45

### KYE28 reduces LPS-induced responses *in vitro* and *ex vivo*

**Figure 2:**
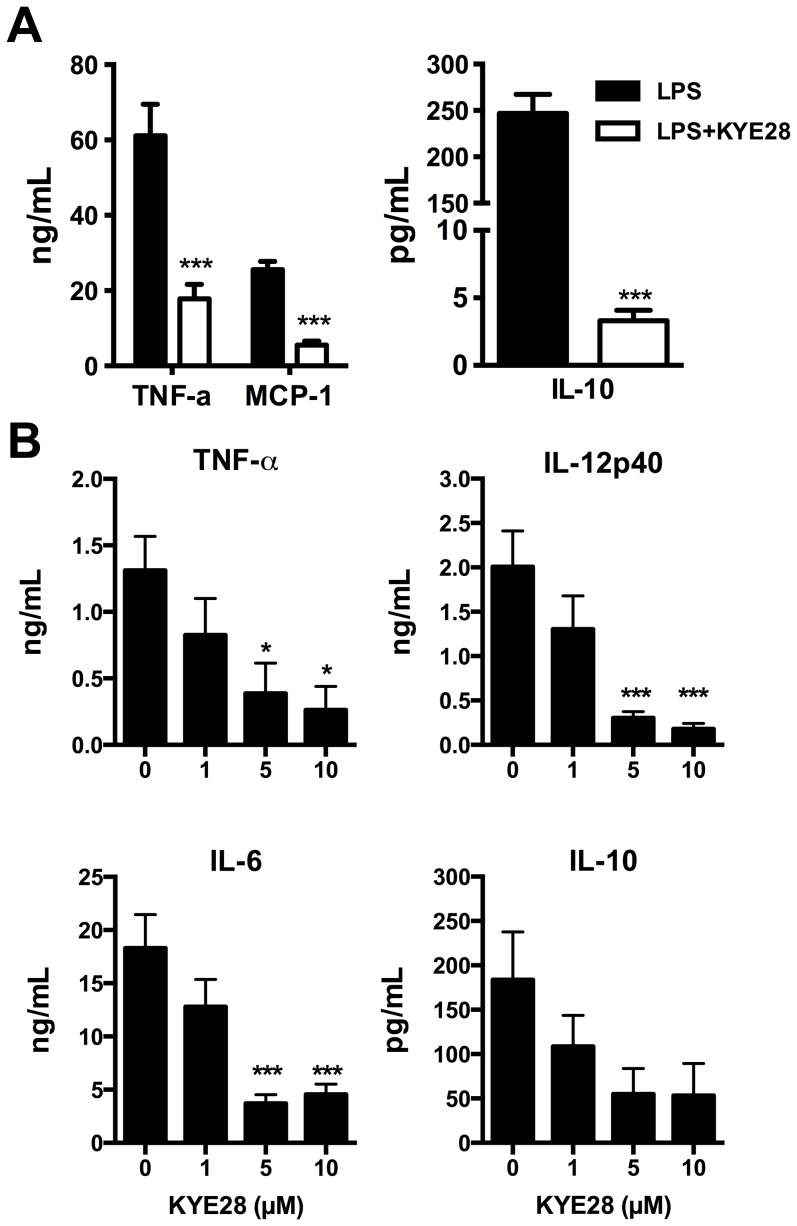
Effects on LPS-induced cytokine responses *in vitro* and ex vivo. (**A**) RAW 264.7 macrophages were stimulated with 10 ng/mL *E. coli* LPS in combination with 10 µM of KYE28. Cytokines were determined in the cell supernatants (n = 3). **(B)** Cytokine analysis of human blood stimulated with 100 ng/mL *E. coli* LPS and indicated concentrations of KYE28 (n = 6).

KYE28-mediated LPS binding and modulation of LPS aggregates were shown to be important factors for LPS-“scavenging” by KYE28 [7], [26]. However, whether these properties also affect LPS-induced cytokine responses in cell-based systems *in vitro* and *ex vivo* remained unknown. Experiments using mouse macrophages showed that 10 µM of KYE28 reduced LPS-induced TNF-α, MCP-1 and IL-10 production by these cells (**Figure 2A**). In agreement, analysis of IL-6, IL-12p40, TNF-α and IL-10 in plasma of human blood *ex vivo* stimulated with 100 ng/mL LPS and KYE28 revealed a dose-dependent inhibition of pro-inflammatory cytokines (**Figure 2B**). Comparable results were obtained with primary monocytes (**data not shown**). Thus, these data show a clear correlation between previous biophysically determined properties on LPS and lipid A binding of KYE28, and its anti-endotoxin effect of KYE28 *in vitro* and *ex vivo*.

### KYE28 interaction with LPS blocks activation of NF-κB/AP1

**Figure 3:**
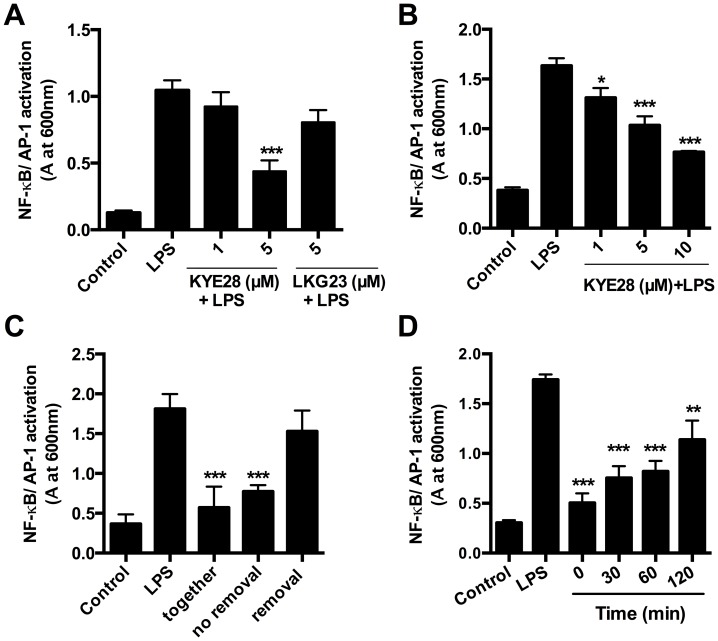
Modulation of NF-κB/AP-1 activation by KYE28. (**A**) Determination of NF-kB/AP-1 activation in supernatants of THP1-X-Blue CD14 cells after stimulation with 100 ng/mL *E. coli* LPS and increasing concentrations of KYE28 (n = 6). The HCII-derived peptide LGK23 (5 µM) was used as control (n = 3). (**B**) NF-kB/AP-1 activity was measured in supernatants of RAW-Blue cells stimulated with 10 ng/mL *E. coli* LPS and increasing concentrations of KYE28 (n = 4). (**C**) RAW-Blue cells stimulated with 10 ng/mL *E. coli* LPS and KYE28 (10 µM). (Together: LPS and peptide added at the same time; no removal: Addition of KYE28 1 h before addition of LPS; removal: Addition of KYE28 for 1 h, removal of the peptide followed by addition of LPS; Control  =  buffer only) (n = 4). (**D**) RAW-Blue cells were stimulated with 10 ng/mL *E. coli* LPS and KYE28 (10 µM) was added after indicated times (n = 5).

Upon LPS binding to the TLR4-MD2 receptor complexes, multiple pathways are initiated which finally lead to the production of for example pro-inflammatory cytokines [37], [38]. Activation of the transcription factors NF-κB and AP-1 is a crucial step within this cascade [37]. Therefore, experiments using NF-κB/AP-1 reporter cell lines were utilized to address effects of KYE28 on NF-κB/AP-1 activation. The data showed that stimulation of either human monocytic reporter cells (THP1-XBlue-CD14) or mouse macrophage reporter cells (RAW-Blue) with *E. coli* LPS clearly initiated NF-κB/AP-1 activation and that this response was dose-dependently reduced in the presence of KYE28 (**Figure 3A and B**). Further, KYE28 also reduced the activation of THP1-XBlue-CD14 cells stimulated with LTA and zymosan, whereas the peptide had no effect on cells stimulated with PGN-EB, PAM_3_CSK_4_ or PMA (**Figure S1**). The HCII-derived control peptide LKG23 had no inhibitory effect (**Figure 3** **, Figure S1**). Experiments using RAW-Blue cells were performed to further investigate the importance of KYE28-LPS interactions for NF-κB/AP-1 activation (**Figure 3C**). Cells were incubated with 10 µM KYE28 for 1 h. Next, the peptide was either removed before LPS addition to the cells (removal), or LPS was added to the peptide-containing medium (no removal). The values were compared to cells stimulated with LPS and KYE28 added at the same time (together). Finally, no significant inhibition of NF-κB/AP-1 activation was detected, when the peptide was removed. Only in settings were peptide and LPS were both present, reduced cell activation was observed (**Figure 3C**). This indicates that KYE28 blocks the LPS-induced responses by efficiently scavenging LPS in the medium. These results are further supported by data showing that KYE28 reduced NF-κB/AP-1 activation when added up to 2 h after LPS stimulation (**Figure 3D**).

### KYE28 effects on eukaryotic cells

As AMPs have been shown to disrupt eukaryotic membranes [26], [39], [40], LDH release was measured to ensure that the detected inhibitory effects were not due to cell death. An increase in the LDH release was observed for the THP1-XBlue-CD14 cells (5 µM KYE28), whereas no significant permeabilisation of RAW-Blue cells was observed with 5-10 µM peptide (**Figure S2, method S1**). Correspondingly, a human keratinocyte cell line (HaCat cells) was permeabilised by the peptide at doses of 6-60 µM, this in a defined serum-free medium (**Figure S3A and C, method S1 and S2)**. It was noted however, that the cathelicidin peptide LL-37 also showed a similar permeabilising activity, illustrating that endogenous AMPs affect eukaryotic cells in defined media conditions. However, in presence of 20% serum no significant LDH release or decrease in cell viability was observed at 60 µM of KYE28 or LL-37 (**Figure S3 B and D, method S1 and S2)**, and in 50% blood (in PBS) the two peptides (at 60 µM) did not cause significant hemolysis (**Figure S4, method S3)**. These results indicate that the extent of permeabilisation is similar to the one observed for LL-37, and is both cell and context dependent, and quenched in plasma or blood.

### KYE28 exerts anti-endotoxic effects *in vivo*

**Figure 4:**
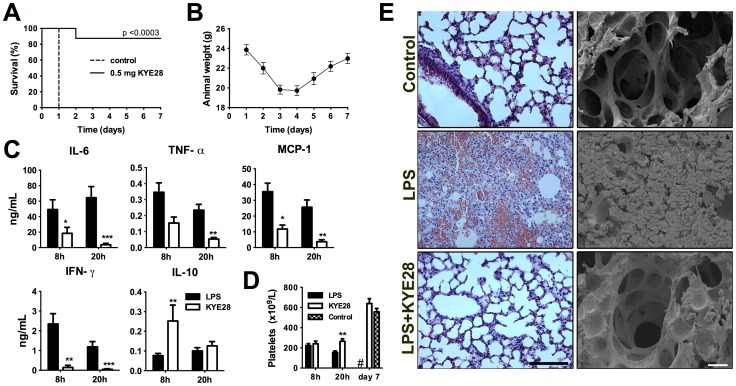
Effects of KYE28 against LPS *in vivo.* (**A-E**) Septic shock in C57BL/6 mice was induced by intraperitoneal (i.p.) injection of *E. coli* LPS (18 mg/kg) followed by i.p. injection of 0.5 mg of KYE28 or buffer 30 min later. (**A**) Survival of the animals challenged with LPS and buffer (Control n = 6) or KYE28 (n = 8) was monitored for 7 days. (**B**) Diagram presenting the weight development during the experiment in (A) for KYE28 treated mice. (**C**) Measurement of cytokines 8 and 20 h after LPS injection in mouse plasma (8 h: LPS n = 12, LPS+KYE28 n = 8; 20 h LPS n = 14, LPS+KYE28 n = 10). (**D**) Number of platelets were determined 8 and 20 h after LPS, as well as in survivors at day 7 (8 h: LPS n = 11, LPS+KYE28 n = 8; 20 h LPS n = 14, LPS+KYE28 n = 7, day 7 LPS+KYE28 n = 7, Control n = 8). (**E**) Lung sections of healthy (Control), LPS treated and LPS+KYE28 treated mice were analysed 20 h after LPS injection. Left panel illustrates representative light microscopy images stained with haematoxylin-eosin (original magnification 20x, scale bar: 100 µM) and the right panel shows representative scanning electron micrographs (scale bar: 20 µM).

Having shown efficient reduction of LPS-induced responses by KYE28 *in vitro* and *ex vivo*, effects of KYE28 were next evaluated in a mouse model of LPS-induced shock. C57BL/6 mice were challenged with 18 mg/kg *E. coli* LPS and treated with either buffer or 0.5 mg KYE28 30 minutes after LPS injection. Ninety percent of KYE28-treated animals survived the experiment, whereas all control animals had to be sacrificed within the first 24 h (**Figure 4A**). Measurements of the weight of KYE28-treated mice indicated that the mice were affected by the LPS challenge, but recovered (**Figure 4B**). In agreement, analyses of cytokines 8 and 20 h after LPS injection yielded significant reductions of pro-inflammatory IL-6, IFN-γ, TNF-α, and MCP-1 for KYE28 treated mice (**Figure 4C**), whereas an increase in IL-10 was observed after 8 h. Thrombocytopenia is a clinical feature of severe sepsis and septic shock [41], but also an indicator for disseminated intravascular coagulation, a detrimental complication seen in sepsis [3], [42]. The analysis of platelet counts revealed a significant decrease in platelets for LPS-challenged mice compared to healthy controls reflecting this clinical parameter (**Figure 4D**). Treatment of animals with KYE28 resulted in less platelet reduction compared to LPS control animals and platelet counts of recovered KYE28-treated mice were not different from healthy controls (**Figure 4D**). Correspondingly, histological and scanning electron microscopy analyses of lungs from LPS and buffer-treated animals showed pulmonary leakage of protein and red blood cells as well as fibrin deposition (**Figure 4E**). These effects were notably suppressed in KYE28-treated animals (**Figure 4E**). Excessive activation of the clotting cascade contributes to the detrimental effects observed during sepsis and septic shock, including platelet consumption and fibrin deposition in the lungs [43]. Considering the observed attenuation of decrease in platelet levels and reduction of fibrin in the lungs after KYE28-treatment, we investigated possible effects of KYE28 on coagulation pathways. Analysis of peptide effects on the activated partial thromboplastin time (aPTT) and prothrombin time (PT) showed that KYE28 impaired the intrinsic pathway (aPTT) of coagulation in human plasma *in vitro* (**Figure S5, method S4**).

In another set of experiments using a lower dose of *E. coli* LPS (12 mg/kg), as low as 50 µg of the peptide were enough to significantly reduce the production of pro-inflammatory cytokines, confirming the significant anti-endotoxin capacity of KYE28 *in vivo* (**Figure S6)**. Moreover, KYE28 also reduced pro-inflammatory cytokines in a similar *in vivo* mouse model of *P. aeruginosa* LPS-induced shock (**Figure S7A**). Similarly to *E. coli* LPS, mice injected with *P. aeruginosa* LPS and treated with KYE28 showed less reduction in platelets compared to controls (**Figure S7B**). This indicates that the effects of KYE28 also apply to endotoxins from other Gram-negative bacteria.

### KYE28 is effective during *Pseudomonas* sepsis

**Figure 5:**
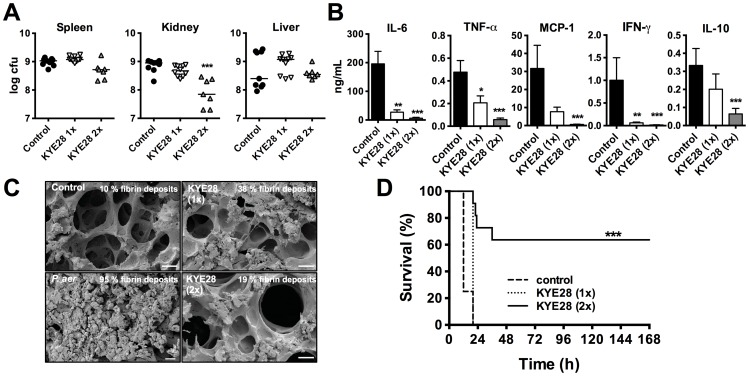
KYE28 modulates cytokines and survival in *Pseudomonas* sepsis. (**A-D**) Mice were challenged with 2×10^9^ cfu/mL *P. aeruginosa* (i.p.) and KYE28 (0.5 mg) was administered s.c. 1 h (KYE28 1x) or 1 and 7 h (KYE28 2x) after injection of bacteria. (**A**) Bacterial counts in the indicated organs were analyzed 12 h post bacteria injection (Control n = 9, KYE28 1x n = 10, KYE28 2x n = 7). (**B**) Twelve hours after bacterial challenge mice were sacrificed and cytokines were measured in plasma (Control n = 13, KYE28 1x n = 11, KYE28 2x n = 10). (**C**) Scanning electron micrographs of representative lung sections 12 h after infection (scale bar: 20 µM). (**D**) After injection of bacteria and treatment with KYE28, status of the animals was monitored for 7 days as described in the methods section (Control n = 16, KYE28 1x n = 10, KYE28 2x n = 11) (***p<0.001, log-rank test).

In order to further explore a potential therapeutic effect of KYE28 in bacterial sepsis, a model employing a clinical isolate of *P. aeruginosa* was used, which was motivated by the fact that infections with *Pseudomonas* species are associated with an increased risk of in-hospital death [4], [5]. To elucidate the effect of KYE28 during disease development, an initial time study was performed (**Figure S8**). The data showed that bacterial levels increased between 4-12 h in the analysed organs (spleen, kidney, and liver). Treatment with the peptide did not reduce bacterial levels, although a tendency for a bacterial reduction among the peptide-treated animals was observed especially in the kidney (**Figure S8A**). Evaluation of cytokines 12 h after bacterial challenge revealed that levels of IL-6, TNF-α, MCP-1 and IFN-γ were all significantly lower in the peptide-treated group (**Figure S8B**). In contrast, the anti-inflammatory IL-10 response was not significantly blocked by the peptide. Based on these results, the effects of one vs. two administrations of KYE28 were analyzed. As above, the results showed that one dose did not significantly reduce bacterial colonies, however repeated treatment caused a moderate, but only for kidney a statistically significant reduction of bacteria (**Figure 5A**). More importantly, however, a two-dose peptide treatment yielded a further decrease of the different pro-inflammatory cytokines (**Figure 5B**), which was accompanied by a clear decrease of inflammatory changes in lungs of peptide-treated animals observed by SEM (**Figure 5C**). Finally, the two-dose peptide treatment resulted in a delay of septic symptoms yielding a significant increase of survival (over 60%) as compared to one-dose and control animals (**Figure 5D**). In this context, it is notable that the peptide alone, when given in doses up to 1 mg per animal, did not cause any apparent signs of adverse reactions in the animals, and the lungs and organs investigated after this treatment looked normal (**data not shown**). Furthermore, the peptide did neither induce any cytokine responses, nor did it affect platelet levels, or coagulation times when given alone (**Figure S9 A-C**).

## Discussion

Research on novel treatments for severe infections and sepsis is an emerging area as illustrated by studies using coagulation proteins or host defense peptides in order to target various aspects of the disease [7], [15], [23], [28], [44]. Along this line, a novel role of proteolytically activated HCII was previously reported in host defense against Gram-negative infections [7]. This lead to the reasoning that antimicrobial epitopes of HCII, might mimic certain host defense functions of the holoprotein [7], [25] and could thus potentially, be utilized as a novel therapeutic molecule in the early stages of infection. The helix D-derived peptide KYE28, the focus of this study, was found to exert antimicrobial effects against Gram-negative bacteria, but also against Gram-positive bacteria as well as *Candida* species. These data were compatible with the previously discovered ability of KYE28 to disrupt artificial liposomes [26], emphasizing that KYE28 possesses critical features of a classical AMP. Previously, a direct interaction of LPS with the holoprotein was demonstrated, mediated by the epitope KYE28 [7], motivating studies on LPS-induced responses *in vitro* and *in vivo*. KYE28 clearly reduced LPS-induced cytokine release, especially that of pro-inflammatory cytokines. The results suggest that the peptide prevents cell activation upstream of NF-κB/AP-1 likely at the cell surface or in the surrounding medium by binding to LPS, which is supported by the results of the pre- and post-incubation experiments as well as by findings showing that KYE28 binds to a similar extent to both LPS and lipid A, and also mediates LPS scavenging on model eukaryotic membranes [26]. Furthermore, KYE28 modulates LPS aggregate structures [26]. The fragmentation and densification of LPS aggregates is dependent on the secondary structure in the peptide/LPS aggregates, and correlates to the anti-endotoxic effect, thus identifying peptide-induced packing transitions in LPS aggregates as key for the anti-endotoxic functionality of KYE28 [26]. Recent studies on host defense peptides have focused on their multiple bioactive properties beyond the direct bacterial killing effects. Indeed, for LL-37 as well as thrombin-derived peptides, it has been argued that the direct antimicrobial function is not the major contributor for beneficial effects during infections *in vivo* [24], [29], [45]. The present results for KYE28 are in line with these previous findings and highlight the relative importance of the anti-inflammatory effect versus the antimicrobial activity *in vivo*. Thus, while KYE28 exerted potent antimicrobial effects *in vitro*, it showed only a moderate activity against bacteria when injected in animals infected by *P. aeruginosa.* In contrast, the reduction of pro-inflammatory responses seems to dominate, as evidenced by reduced cytokine levels as well as improved lung status.

Inflammation and coagulation are interlinked processes [8] and the activation of both by LPS or bacteria lead amongst others to fibrin deposition in the microvasculature as observed in the lungs from mice subjected to LPS-mediated shock and *P. aeruginosa* infection (**Figure 4** **and** **5**). As a result, microvascular thrombosis contributes to promotion of organ dysfunction. Moreover, excessive contact activation results in the release of the pro-inflammatory peptide bradykinin and a subsequent induction of inflammatory reactions, which contribute to serious complications such as hypotension and vascular leakage [46], [47]. The observed reductions in lung leakage and fibrin deposition, are consistent with studies showing that IL-6 is an important factor for inflammation-driven coagulation [8], [48], as IL-6 was significantly blocked by KYE28. Furthermore, it is possible that KYE28, by reducing the activation of the contact system, may mediate reductions in lung fibrin deposition and platelet levels, respectively, as previously noted for an endogenous multifunctional host defense peptide of thrombin [24].

An interesting observation was also that the overall reduction of pro-inflammatory cytokines in animals infected for 12 h was not complete, but seemed to be reduced to those levels found during the initial phases of sepsis development observed after 4–8 h. Thus, the action of KYE28 contrasts to other substances with more marked or even complete inhibitory action on LPS-signaling, e. g. TLR-4 inhibitors [49]. From a clinical perspective, dampening of the initial pro-inflammatory response by KYE28-treatment, especially reducing IL-6, and MCP-1, which have been associated with organ dysfunction, severity of the disease and mortality [50]–[53], may aid in preventing the development of the detrimental “cytokine storm” and its consequences seen in sepsis [51]. Thus, complete blocking of inflammatory responses may be negative for the proper resolution of an infection *in vivo*, a reasoning compatible with the observation that TLR-4-deficient animals are much less sensitive to endotoxins, while being highly prone to infections [54]. Cleary, both dose and administration times are crucial. For example, in initial tests, KYE28, when administrated in the LPS model at later time points (2–4 h post LPS injection) did not reverse the endotoxin mediated mortality, although an increase was noted in the survival time when KYE28 was injected after 2 hours (not shown). Nevertheless, in the *Pseudomonas* sepsis model, where the peptide was given subcutaneously 1 and 7 hours after intraperitoneal *Pseudomonas* infection (avoiding compartmentalization of bacteria and peptide together) significant anti-inflammatory effects as well as mortality reductions were observed. These observations illustrate that a “fast” model, where systemic LPS-mediated activations occur within a short time frame, is less suitable for delayed treatment studies, whereas the latter *Pseudomonas* sepsis model clearly demonstrated a therapeutic potential of delayed peptide treatment. Although beyond the scope of the present study, future therapeutical and developmental studies must address not only peptide pharmacokinetics and toxicity in more detail, but also effects in more complex models, such as the cecum ligation and puncture model of polymicrobial sepsis.

Finally, it must also be stressed that the activities of KYE28 may not necessarily reflect all possible activities mediated by proteolytically activated HCII. Thus, it is possible that the bacterial binding and LPS-interactions that are mediated by this helix D region, or its counterpart on helix A, may be complemented by other actions of distant structural motifs in HCII. Nevertheless, the present data indicate, that the current strategy of selecting a functional epitope of HCII may have potential therapeutic benefits due to a less complex mode of action and easier production of the peptide, while maintaining the endogenous character of the host response.

## Supporting Information

Figure S1 **Effects of KYE28 on various cell agonists.** THP1-X-Blue CD14 cells were stimulated either with 1 µg/mL lipoteichoic acid (LTA), 10 µg/mL Zymosan, 1 µg/mL *E. coli-*derived peptidoglycan (PGN-EB), 20 ng/mL PAM_3_CSK_4_ or 100 ng/mL PMA together with 5 µM of KYE28 or the control peptide LKG23. NF-κB/AP-1 activation was determined after over night incubation in cell supernatants (n = 3).
(TIF)

Figure S2 **Influence of KYE28 on eukaryotic membranes.** (**A**) THP1-X-Blue CD14 cells were stimulated over night either with 100 ng/mL *E. coli* LPS together with the indicated concentration of KYE28 (LPS) or only KYE28 (no LPS). LDH release was determined in cell supernatants (n = 4). (**B**) Same assay as in (A), but RAW-Blue cells were used (n = 4).
(TIF)

Figure S3 **Evaluation of toxic effects of KYE28 on HaCat cells.** (**A**) LDH release of HaCat cells grown in serum-free medium was measured after over night incubation with indicated concentrations of KYE28 and LL-37 (n = 4). (**B**) Same as in (A), but in the presence of 20% human serum and 60 µM of the peptides were used (n = 3). (**C**) HaCat cells grown in serum-free medium were incubated over night with indicated concentrations of the peptides. Cell viability was determined using the MTT assay (n = 4). (**D**) Same as in (C), in the presence of 20% human serum and usage of 60 µM of the peptides (n = 3).
(TIF)

Figure S4 **Evaluation of hemolytic effects of KYE28 in blood.** Hemolysis in 50% human citrate-blood (diluted 1∶1 in PBS) in presence of KYE28 (60 µM) is shown. Hemolysis was assessed after 1 hour. LL-37 is shown for comparison (n = 3).
(TIF)

Figure S5 **Effects of KYE28 on coagulation** ***in vitro.*** Fresh human citrate plasma was incubated with buffer (Control) or 20 µM of KYE28 before the activated partial thromboplastin time (aPTT), prothrombin time (PT) and the thrombin clotting time (TCT) were determined (n = 2).
(TIF)

Figure S6 **Dose-dependent effects of KYE28 in a LPS model** ***in vivo.*** C57BL/6 mice were challenged with 12 mg/kg *E. coli* LPS (i.p.) and treated after 30 min with indicated amounts of KYE28 (i.p.). Cytokines were evaluated 20 h post-LPS injection in the plasma (no peptide n = 8; KYE28 treated n = 5/group).
(TIF)

Figure S7 **Effects of KYE28 in a** ***Pseudomonas*** **LPS model** ***in vivo.*** (**A-B**) C57BL/6 mice were treated with 36 mg/kg *Pseudomonas* LPS (i.p.) and treated with buffer or 0.5 mg KYE28 (i.p.). Twenty hours post-LPS injection, blood was taken and analyzed for (**A**) indicated cytokines (P-LPS n = 8, P-LPS+KYE28 n = 10) and (**B**) platelet counts (Control n = 8, P-LPS n = 6, P-LPS+KYE28 n = 9).
(TIF)

Figure S8 **Evaluation of KYE28 treatment in a** ***Pseudomonas*** **infection model** ***in vivo.*** (**A-B**) C57BL/6 mice were infected i.p. with 2×10^9^ cfu/mL *P. aeruginosa* 15159. KYE28 (0.5 mg) was subcutaneously injected one h after infection. (**A**) Bacterial counts in the indicated organs were analyzed after a time period of 4, 8, and 12 h. (Control 4 h n = 5, 8 h n =  5, 12 h n = 4; KYE28 n = 7/group). (**B**) In parallel, the indicated cytokines were analyzed in plasma from those mice (Control n = 9, KYE28 n = 11).
(TIF)

Figure S9 **Analysis of KYE28 given alone.** (**A-C**) Subcutaneous administration of 1 mg KYE28 or buffer (Control). Treatment was repeated 6 h post-injection and indicated parameters analyzed 12 h post-injection. (**A**) Cytokines determined in plasma are presented with the corresponding detection limits of the assay. (**B**) Determination of platelets. (**C**) Measurement of activated partial thromboplastin time (aPTT) and prothrombin time (PT) in mouse plasma (n = 6/group).
(TIF)

Method S1 **Lactate dehydrogenase (LDH) assay.**
(DOCX)

Method S2 **Cell viability assay (MTT assay).**
(DOCX)

Method S3 **Hemolysis assay.**
(DOCX)

Method S4 **Coagulation assay.**
(DOCX)

## References

[1] French GL (2010) The continuing crisis in antibiotic resistance. Int J Antimicrob Agents 36 Suppl 3S3–7. 10.1016/S0924-8579(10)70003-0 21129629

[2] Rossolini GM, Mantengoli E (2008) Antimicrobial resistance in Europe and its potential impact on empirical therapy. Clin Microbiol Infect 14 Suppl 62–8. 10.1111/j.1469-0691.2008.02126.x 19040461

[3] Angus DC, van der Poll T (2013) Severe sepsis and septic shock. N Engl J Med 369: 840–851. 23984731 10.1056/NEJMra1208623

[4] Vincent JL, Rello J, Marshall J, Silva E, Anzueto A, et al (2009) International study of the prevalence and outcomes of infection in intensive care units. JAMA 302: 2323–2329. 19952319 10.1001/jama.2009.1754

[5] Vincent JL, Sakr Y, Sprung CL, Ranieri VM, Reinhart K, et al (2006) Sepsis in European intensive care units: results of the SOAP study. Crit Care Med 34: 344–353. 16424713 10.1097/01.ccm.0000194725.48928.3a

[6] Martin GS, Mannino DM, Eaton S, Moss M (2003) The epidemiology of sepsis in the United States from 1979 through 2000. N Engl J Med 348: 1546–1554. 12700374 10.1056/NEJMoa022139

[7] Kalle M, Papareddy P, Kasetty G, Tollefsen DM, Malmsten M, et al (2013) Proteolytic activation transforms heparin cofactor II into a host defense molecule. J Immunol 190: 6303–6310. 23656734 10.4049/jimmunol.1203030 PMC3677170

[8] Levi M, van der Poll T (2010) Inflammation and coagulation. Crit Care Med 38: S26–34. 20083910 10.1097/CCM.0b013e3181c98d21

[9] Wiedermann CJ, Hoffmann JN, Juers M, Ostermann H, Kienast J, et al (2006) High-dose antithrombin III in the treatment of severe sepsis in patients with a high risk of death: efficacy and safety. Crit Care Med 34: 285–292. 16424704 10.1097/01.ccm.0000194731.08896.99

[10] Abraham E, Reinhart K, Opal S, Demeyer I, Doig C, et al (2003) Efficacy and safety of tifacogin (recombinant tissue factor pathway inhibitor) in severe sepsis: a randomized controlled trial. JAMA 290: 238–247. 12851279 10.1001/jama.290.2.238

[11] Laterre PF, Opal SM, Abraham E, LaRosa SP, Creasey AA, et al (2009) A clinical evaluation committee assessment of recombinant human tissue factor pathway inhibitor (tifacogin) in patients with severe community-acquired pneumonia. Crit Care 13: R36. 19284881 10.1186/cc7747 PMC2689471

[12] Van Den Boogaard FE, Brands X, Schultz MJ, Levi M, Roelofs JJ, et al (2011) Recombinant human tissue factor pathway inhibitor exerts anticoagulant, anti-inflammatory and antimicrobial effects in murine pneumococcal pneumonia. J Thromb Haemost 9: 122–132. 21029363 10.1111/j.1538-7836.2010.04089.x

[13] Marti-Carvajal AJ, Sola I, Lathyris D, Cardona AF (2012) Human recombinant activated protein C for severe sepsis. Cochrane Database Syst Rev 3: CD004388. 10.1002/14651858.CD004388.pub5 22419295

[14] Abraham E, Reinhart K, Svoboda P, Seibert A, Olthoff D, et al (2001) Assessment of the safety of recombinant tissue factor pathway inhibitor in patients with severe sepsis: a multicenter, randomized, placebo-controlled, single-blind, dose escalation study. Crit Care Med 29: 2081–2089. 11700399 10.1097/00003246-200111000-00007

[15] Hancock RE, Sahl HG (2006) Antimicrobial and host-defense peptides as new anti-infective therapeutic strategies. Nat Biotechnol 24: 1551–1557. 17160061 10.1038/nbt1267

[16] Fox JL (2013) Antimicrobial peptides stage a comeback. Nat Biotechnol 31: 379–382. 23657384 10.1038/nbt.2572

[17] Pasupuleti M, Schmidtchen A, Malmsten M (2012) Antimicrobial peptides: key components of the innate immune system. Crit Rev Biotechnol 32: 143–171. 22074402 10.3109/07388551.2011.594423

[18] Lai Y, Gallo RL (2009) AMPed up immunity: how antimicrobial peptides have multiple roles in immune defense. Trends Immunol 30: 131–141. 19217824 10.1016/j.it.2008.12.003 PMC2765035

[19] Harder J, Glaser R, Schroder JM (2007) Human antimicrobial proteins effectors of innate immunity. J Endotoxin Res 13: 317–338. 18182460 10.1177/0968051907088275

[20] Zasloff M (2002) Antimicrobial peptides of multicellular organisms. Nature 415: 389–395. 11807545 10.1038/415389a

[21] Tossi A, Sandri L, Giangaspero A (2000) Amphipathic, alpha-helical antimicrobial peptides. Biopolymers 55: 4–30. 10931439 10.1002/1097-0282(2000)55:1<4::AID-BIP30>3.0.CO;2-M

[22] Bowdish DM, Davidson DJ, Hancock RE (2006) Immunomodulatory properties of defensins and cathelicidins. Curr Top Microbiol Immunol 306: 27–66. 16909917 10.1007/3-540-29916-5_2 PMC7121507

[23] Nijnik A, Madera L, Ma S, Waldbrook M, Elliott MR, et al (2010) Synthetic cationic peptide IDR-1002 provides protection against bacterial infections through chemokine induction and enhanced leukocyte recruitment. J Immunol 184: 2539–2550. 20107187 10.4049/jimmunol.0901813

[24] Kalle M, Papareddy P, Kasetty G, Morgelin M, van der Plas MJ, et al (2012) Host defense peptides of thrombin modulate inflammation and coagulation in endotoxin-mediated shock and Pseudomonas aeruginosa sepsis. PLoS One 7: e51313. 23272096 10.1371/journal.pone.0051313 PMC3521733

[25] Papareddy P, Kalle M, Singh S, Morgelin M, Schmidtchen A, et al (2014) An antimicrobial helix A-derived peptide of heparin cofactor II blocks endotoxin responses in vivo. Biochim Biophys Acta 1838: 1225–1234. 24522010 10.1016/j.bbamem.2014.01.026

[26] Singh S, Papareddy P, Kalle M, Schmidtchen A, Malmsten M (2013) Importance of lipopolysaccharide aggregate disruption for the anti-endotoxic effects of heparin cofactor II peptides. Biochim Biophys Acta 1828: 2709–2719. 23806651 10.1016/j.bbamem.2013.06.015

[27] Vandamme D, Landuyt B, Luyten W, Schoofs L (2012) A comprehensive summary of LL-37, the factotum human cathelicidin peptide. Cell Immunol 280: 22–35. 23246832 10.1016/j.cellimm.2012.11.009

[28] Papareddy P, Rydengard V, Pasupuleti M, Walse B, Morgelin M, et al (2010) Proteolysis of human thrombin generates novel host defense peptides. PLoS Pathog 6: e1000857. 20421939 10.1371/journal.ppat.1000857 PMC2858699

[29] Mookherjee N, Brown KL, Bowdish DM, Doria S, Falsafi R, et al (2006) Modulation of the TLR-mediated inflammatory response by the endogenous human host defense peptide LL-37. J Immunol 176: 2455–2464. 16456005 10.4049/jimmunol.176.4.2455

[30] Lehrer RI, Rosenman M, Harwig SS, Jackson R, Eisenhauer P (1991) Ultrasensitive assays for endogenous antimicrobial polypeptides. J Immunol Methods 137: 167–173. 1901580 10.1016/0022-1759(91)90021-7

[31] Papareddy P, Kalle M, Kasetty G, Morgelin M, Rydengard V, et al (2010) C-terminal peptides of tissue factor pathway inhibitor are novel host defense molecules. J Biol Chem 285: 28387–28398. 20592020 10.1074/jbc.M110.127019 PMC2934703

[32] Wiegand I, Hilpert K, Hancock RE (2008) Agar and broth dilution methods to determine the minimal inhibitory concentration (MIC) of antimicrobial substances. Nat Protoc 3: 163–175. 18274517 10.1038/nprot.2007.521

[33] Sonesson A, Kasetty G, Olin AI, Malmsten M, Morgelin M, et al (2011) Thymic stromal lymphopoietin exerts antimicrobial activities. Exp Dermatol 20: 1004–1010. 22092577 10.1111/j.1600-0625.2011.01391.x

[34] Kasetty G, Papareddy P, Kalle M, Rydengard V, Walse B, et al (2011) The C-terminal sequence of several human serine proteases encodes host defense functions. J Innate Immun 3: 471–482. 21576923 10.1159/000327016

[35] Pasupuleti M, Walse B, Svensson B, Malmsten M, Schmidtchen A (2008) Rational design of antimicrobial C3a analogues with enhanced effects against Staphylococci using an integrated structure and function-based approach. Biochemistry 47: 9057–9070. 18690701 10.1021/bi800991e

[36] Fritsche TR, Rhomberg PR, Sader HS, Jones RN (2008) Antimicrobial activity of omiganan pentahydrochloride tested against contemporary bacterial pathogens commonly responsible for catheter-associated infections. J Antimicrob Chemother 61: 1092–1098. 18310135 10.1093/jac/dkn074

[37] Takeda K, Akira S (2004) TLR signaling pathways. Semin Immunol 16: 3–9. 14751757 10.1016/j.smim.2003.10.003

[38] Jerala R (2007) Structural biology of the LPS recognition. Int J Med Microbiol 297: 353–363. 17481951 10.1016/j.ijmm.2007.04.001

[39] Kasetty G, Papareddy P, Kalle M, Rydengard V, Morgelin M, et al (2011) Structure-activity studies and therapeutic potential of host defense peptides of human thrombin. Antimicrob Agents Chemother 55: 2880–2890. 21402837 10.1128/AAC.01515-10 PMC3101415

[40] Lehrer RI, Lichtenstein AK, Ganz T (1993) Defensins: antimicrobial and cytotoxic peptides of mammalian cells. Annu Rev Immunol 11: 105–128. 8476558 10.1146/annurev.iy.11.040193.000541

[41] Dellinger RP, Levy MM, Rhodes A, Annane D, Gerlach H, et al (2013) Surviving Sepsis Campaign: international guidelines for management of severe sepsis and septic shock, 2012. Intensive Care Med 39: 165–228. 23361625 10.1007/s00134-012-2769-8 PMC7095153

[42] Levi M, Ten Cate H (1999) Disseminated intravascular coagulation. N Engl J Med 341: 586–592. 10451465 10.1056/NEJM199908193410807

[43] Levi M, de Jonge E, van der Poll T, ten Cate H (1999) Disseminated intravascular coagulation. Thromb Haemost 82: 695–705. 10605770

[44] Schuerholz T, Brandenburg K, Marx G (2012) Antimicrobial peptides and their potential application in inflammation and sepsis. Crit Care 16: 207. 22429567 10.1186/cc11220 PMC3681352

[45] Bowdish DM, Davidson DJ, Lau YE, Lee K, Scott MG, et al (2005) Impact of LL-37 on anti-infective immunity. J Leukoc Biol 77: 451–459. 15569695 10.1189/jlb.0704380

[46] Herwald H, Morgelin M, Olsen A, Rhen M, Dahlback B, et al (1998) Activation of the contact-phase system on bacterial surfaces—a clue to serious complications in infectious diseases. Nat Med 4: 298–302. 9500602 10.1038/nm0398-298

[47] Oehmcke S, Herwald H (2010) Contact system activation in severe infectious diseases. J Mol Med (Berl) 88: 121–126. 20232512 10.1007/s00109-009-0564-y

[48] Dhainaut JF, Shorr AF, Macias WL, Kollef MJ, Levi M, et al (2005) Dynamic evolution of coagulopathy in the first day of severe sepsis: relationship with mortality and organ failure. Crit Care Med 33: 341–348. 15699837 10.1097/01.ccm.0000153520.31562.48

[49] Sha T, Sunamoto M, Kitazaki T, Sato J, Ii M, et al (2007) Therapeutic effects of TAK-242, a novel selective Toll-like receptor 4 signal transduction inhibitor, in mouse endotoxin shock model. Eur J Pharmacol 571: 231–239. 17632100 10.1016/j.ejphar.2007.06.027

[50] Bozza FA, Salluh JI, Japiassu AM, Soares M, Assis EF, et al (2007) Cytokine profiles as markers of disease severity in sepsis: a multiplex analysis. Crit Care 11: R49. 17448250 10.1186/cc5783 PMC2206478

[51] de Jong HK, van der Poll T, Wiersinga WJ (2010) The systemic pro-inflammatory response in sepsis. J Innate Immun 2: 422–430. 20530955 10.1159/000316286

[52] Faix JD (2013) Biomarkers of sepsis. Crit Rev Clin Lab Sci 50: 23–36. 23480440 10.3109/10408363.2013.764490 PMC3613962

[53] Patel RT, Deen KI, Youngs D, Warwick J, Keighley MR (1994) Interleukin 6 is a prognostic indicator of outcome in severe intra-abdominal sepsis. Br J Surg 81: 1306–1308. 7953393 10.1002/bjs.1800810914

[54] Albiger B, Dahlberg S, Henriques-Normark B, Normark S (2007) Role of the innate immune system in host defence against bacterial infections: focus on the Toll-like receptors. J Intern Med 261: 511–528. 17547708 10.1111/j.1365-2796.2007.01821.x

