# Supplementary figures and images for: A Peptide of Heparin Cofactor II Inhibits Endotoxin-Mediated Shock and Invasive Pseudomonas aeruginosa Infection
Source: PLoS One. 2014 Jul 21;9(7):e102577. doi: 10.1371/journal.pone.0102577 (PMC4105479; doi:10.1371/journal.pone.0102577)

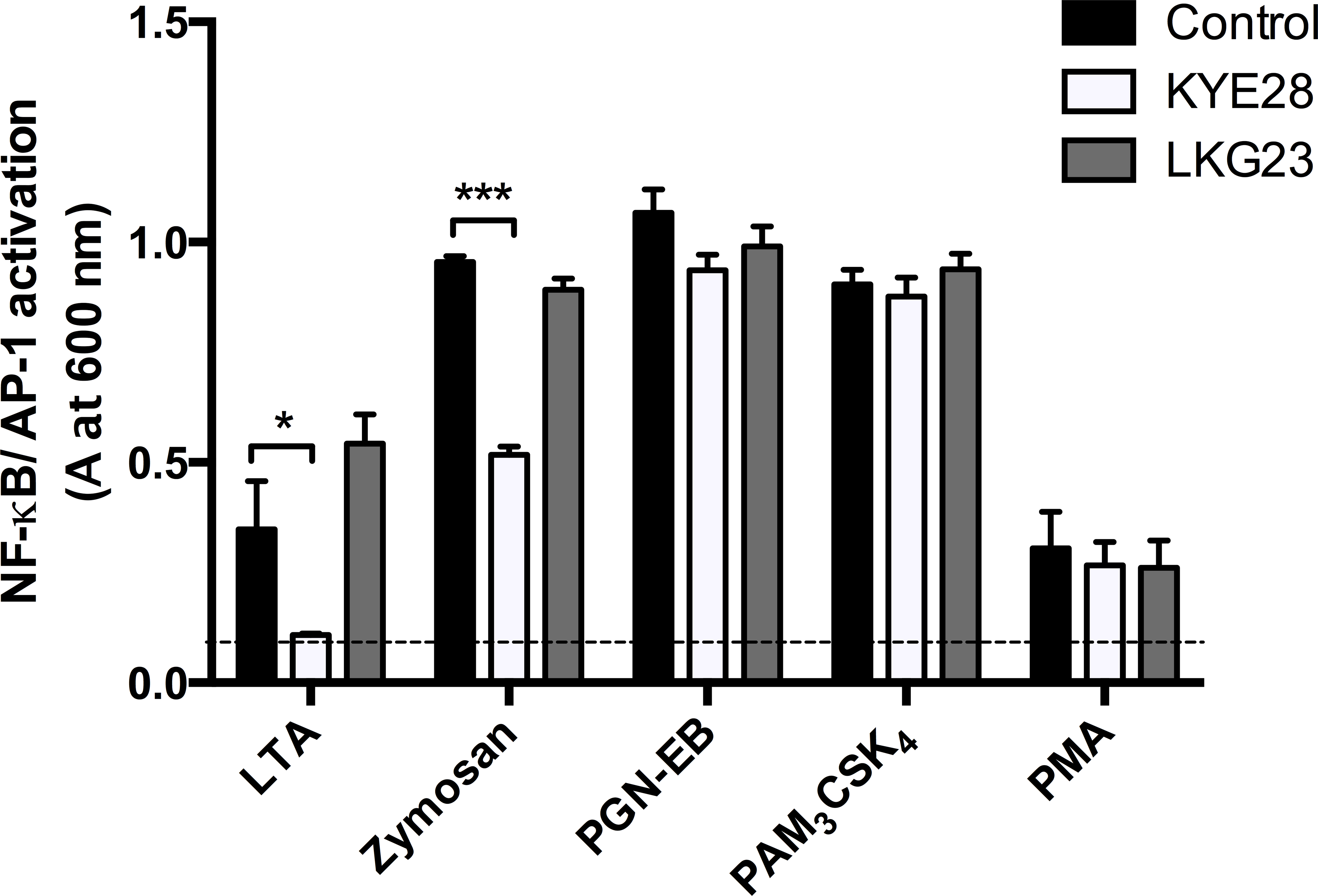

Supplement: Figure S1 — Effects of KYE28 on various cell agonists. THP1-X-Blue CD14 cells were stimulated either with 1 µg/mL lipoteichoic acid (LTA), 10 µg/mL Zymosan, 1 µg/mL E. coli-derived peptidoglycan (PGN-EB), 20 ng/mL PAM3CSK4 or 100 ng/mL PMA together with 5 µM of KYE28 or the control peptide LKG23. NF-κB/AP-1 activation was determined after over night incubation in cell supernatants (n = 3). (TIF) [file pone.0102577.s001.tif]

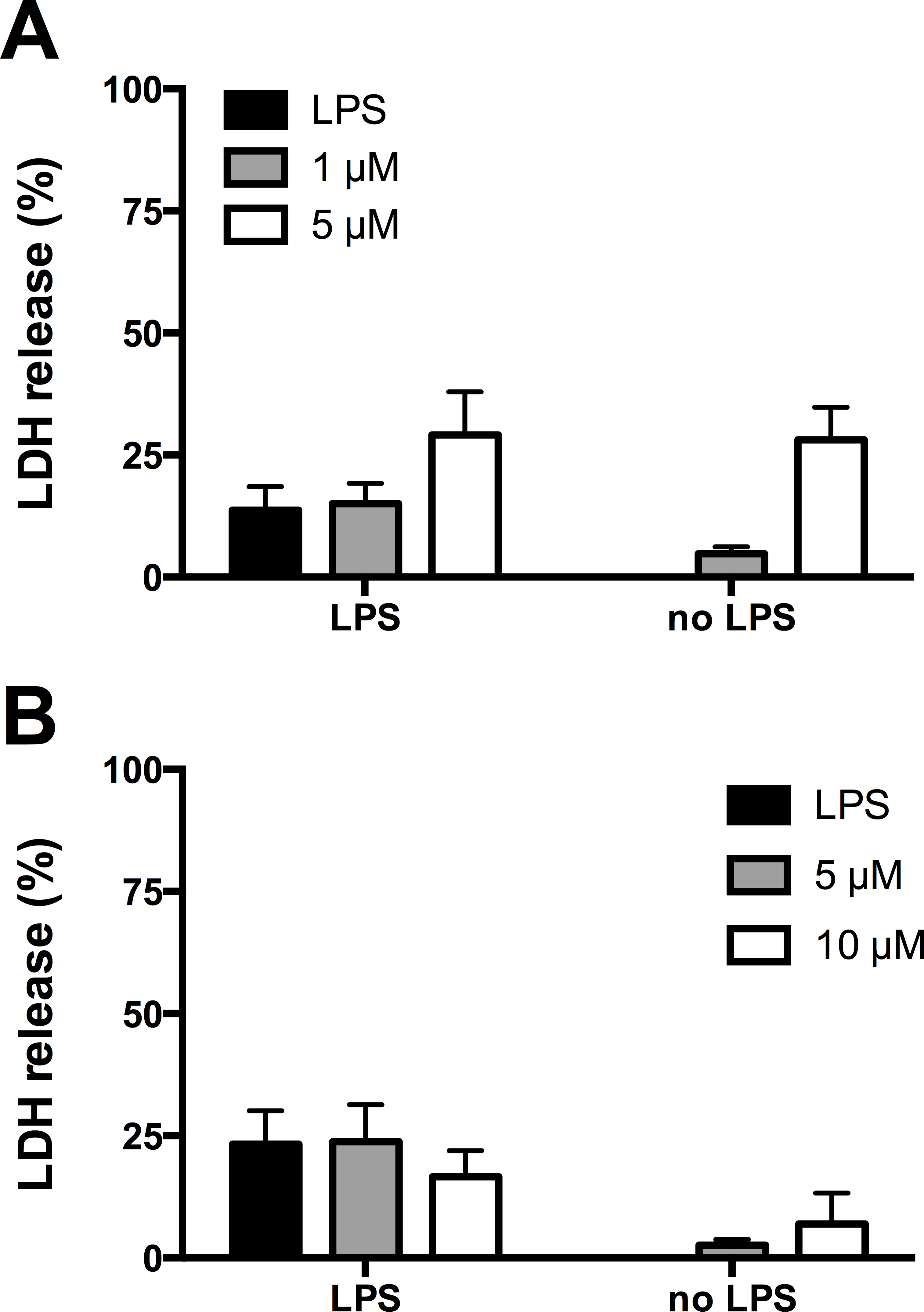

Supplement: Figure S2 — Influence of KYE28 on eukaryotic membranes. (A) THP1-X-Blue CD14 cells were stimulated over night either with 100 ng/mL E. coli LPS together with the indicated concentration of KYE28 (LPS) or only KYE28 (no LPS). LDH release was determined in cell supernatants (n = 4). (B) Same assay as in (A), but RAW-Blue cells were used (n = 4). (TIF) [file pone.0102577.s002.tif]

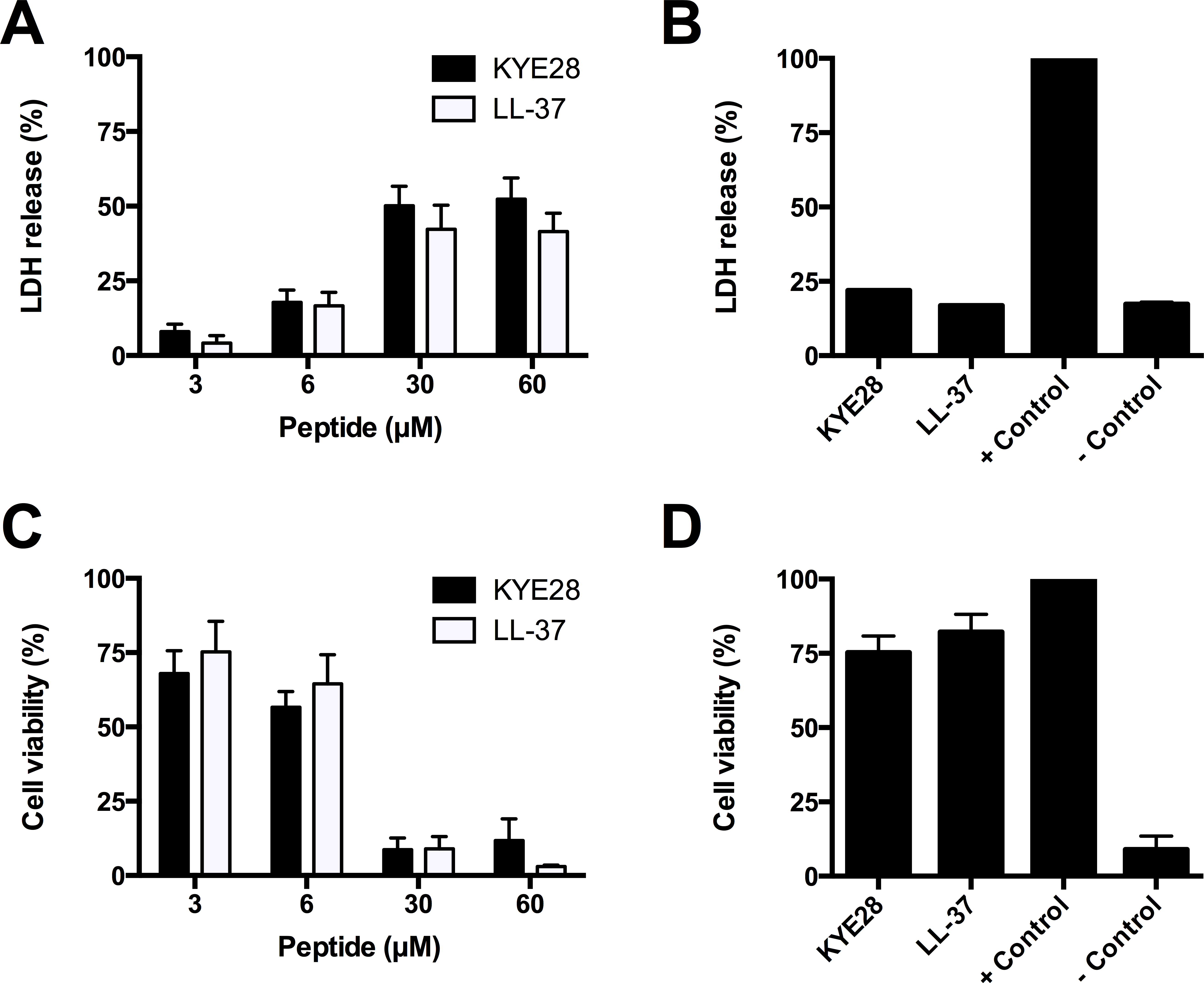

Supplement: Figure S3 — Evaluation of toxic effects of KYE28 on HaCat cells. (A) LDH release of HaCat cells grown in serum-free medium was measured after over night incubation with indicated concentrations of KYE28 and LL-37 (n = 4). (B) Same as in (A), but in the presence of 20% human serum and 60 µM of the peptides were used (n = 3). (C) HaCat cells grown in serum-free medium were incubated over night with indicated concentrations of the peptides. Cell viability was determined using the MTT assay (n = 4). (D) Same as in (C), in the presence of 20% human serum and usage of 60 µM of the peptides (n = 3). (TIF) [file pone.0102577.s003.tif]

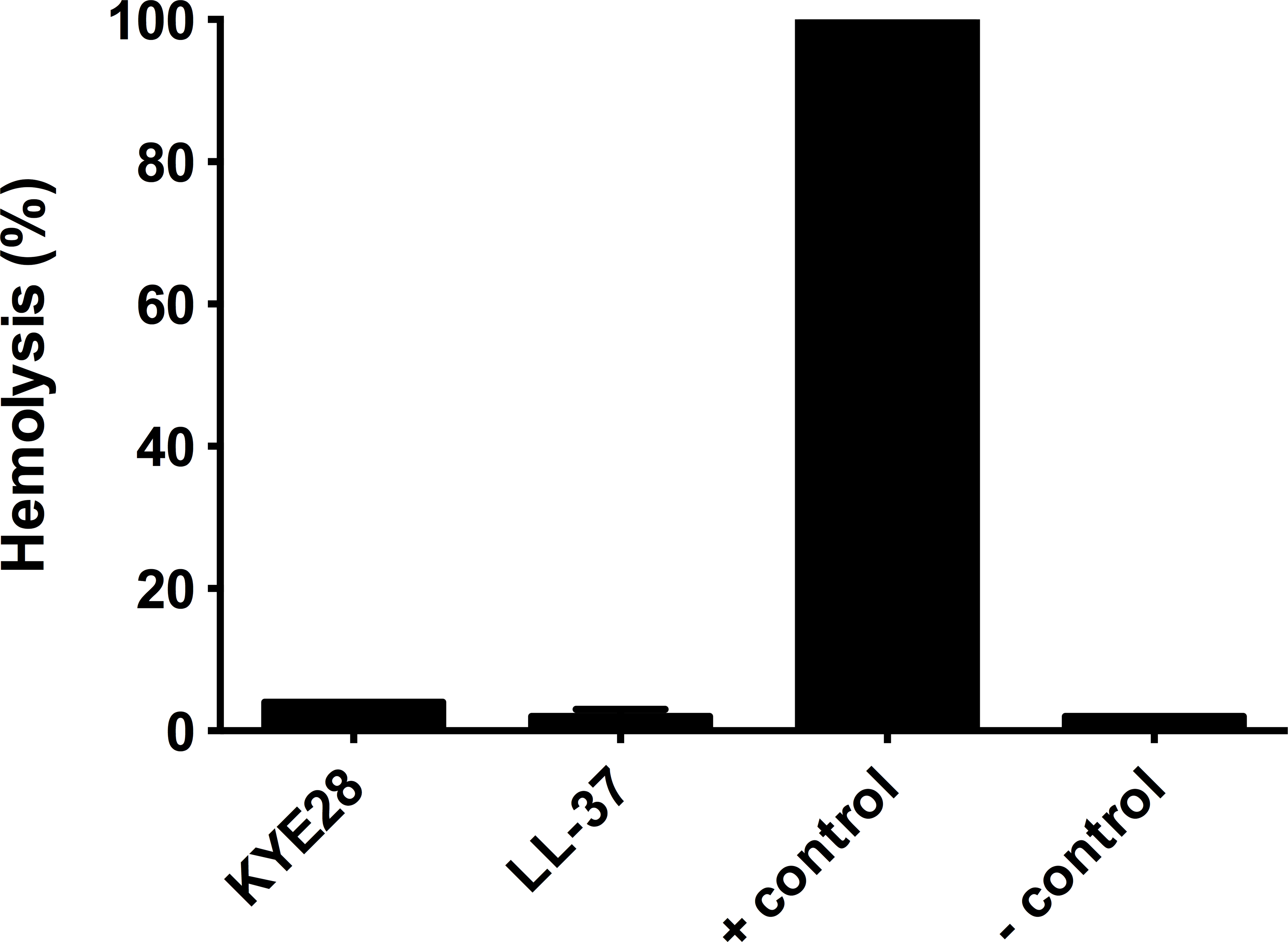

Supplement: Figure S4 — Evaluation of hemolytic effects of KYE28 in blood. Hemolysis in 50% human citrate-blood (diluted 1∶1 in PBS) in presence of KYE28 (60 µM) is shown. Hemolysis was assessed after 1 hour. LL-37 is shown for comparison (n = 3). (TIF) [file pone.0102577.s004.tif]

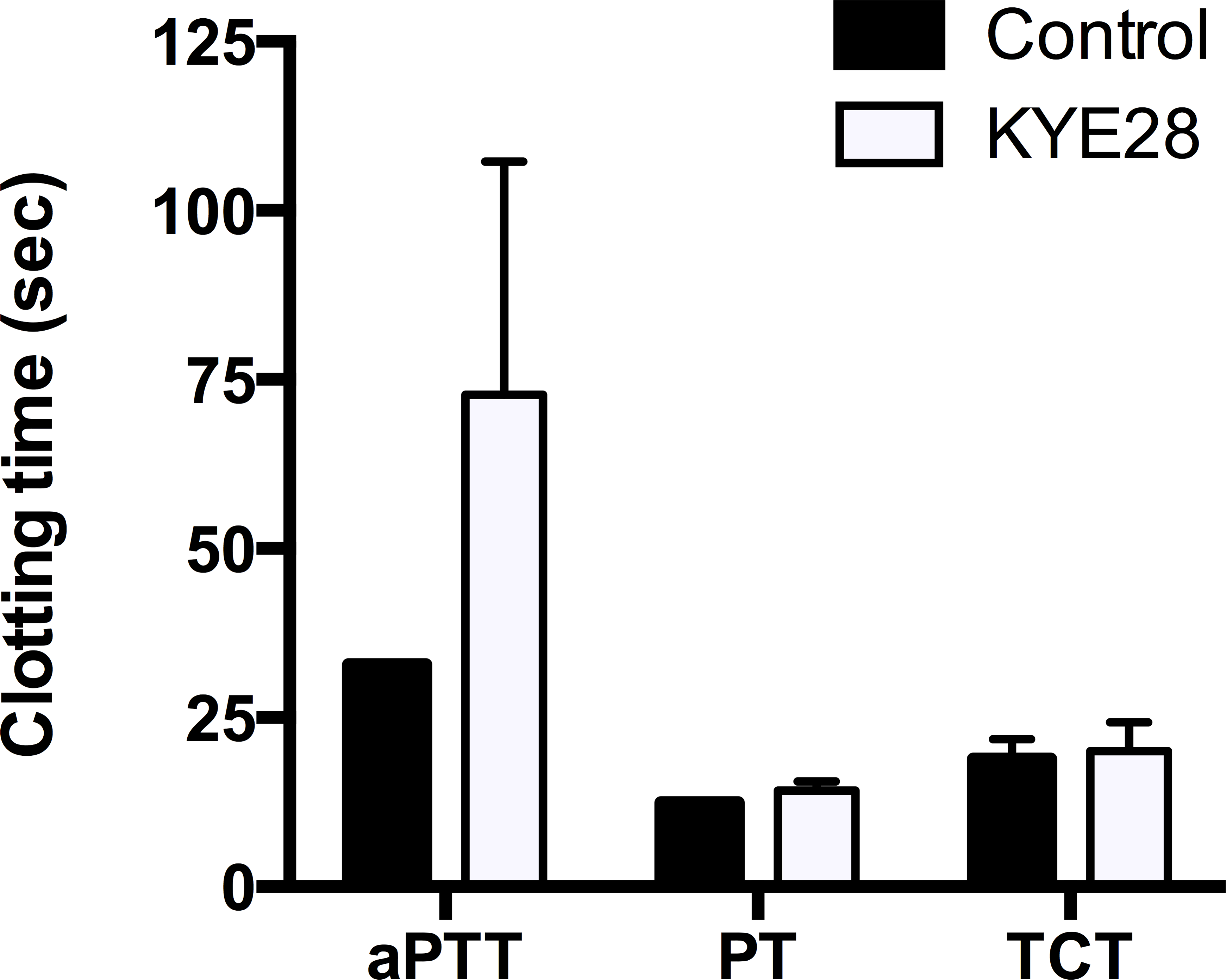

Supplement: Figure S5 — Effects of KYE28 on coagulation in vitro. Fresh human citrate plasma was incubated with buffer (Control) or 20 µM of KYE28 before the activated partial thromboplastin time (aPTT), prothrombin time (PT) and the thrombin clotting time (TCT) were determined (n = 2). (TIF) [file pone.0102577.s005.tif]

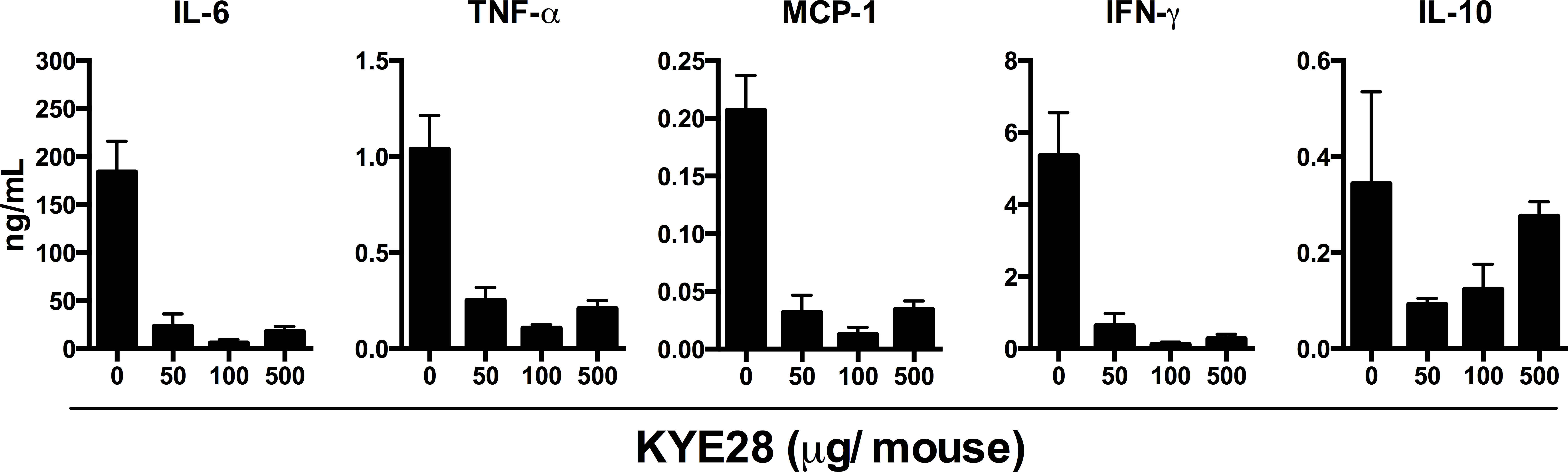

Supplement: Figure S6 — Dose-dependent effects of KYE28 in a LPS model in vivo. C57BL/6 mice were challenged with 12 mg/kg E. coli LPS (i.p.) and treated after 30 min with indicated amounts of KYE28 (i.p.). Cytokines were evaluated 20 h post-LPS injection in the plasma (no peptide n = 8; KYE28 treated n = 5/group). (TIF) [file pone.0102577.s006.tif]

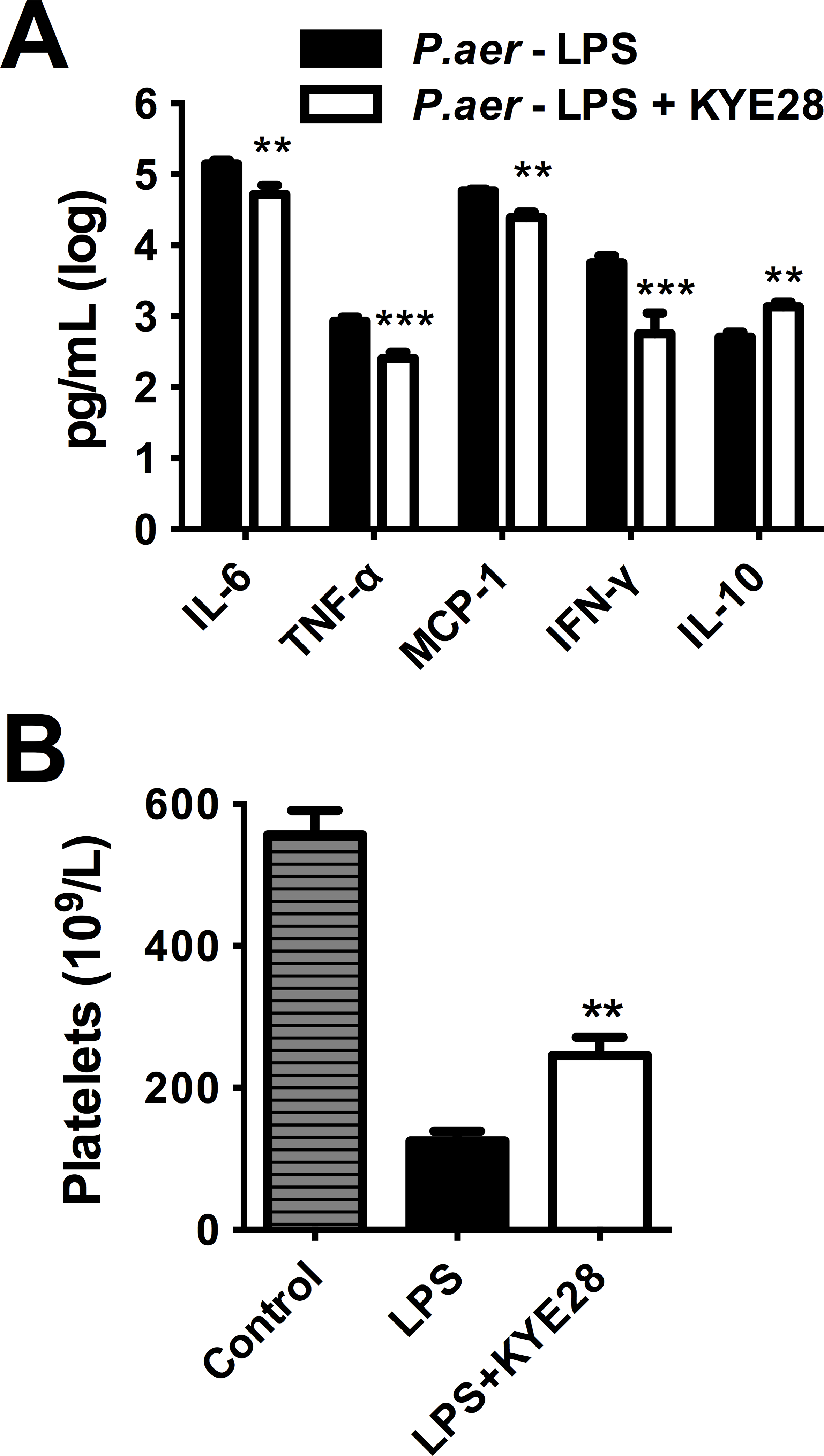

Supplement: Figure S7 — Effects of KYE28 in a Pseudomonas LPS model in vivo. (A-B) C57BL/6 mice were treated with 36 mg/kg Pseudomonas LPS (i.p.) and treated with buffer or 0.5 mg KYE28 (i.p.). Twenty hours post-LPS injection, blood was taken and analyzed for (A) indicated cytokines (P-LPS n = 8, P-LPS+KYE28 n = 10) and (B) platelet counts (Control n = 8, P-LPS n = 6, P-LPS+KYE28 n = 9). (TIF) [file pone.0102577.s007.tif]

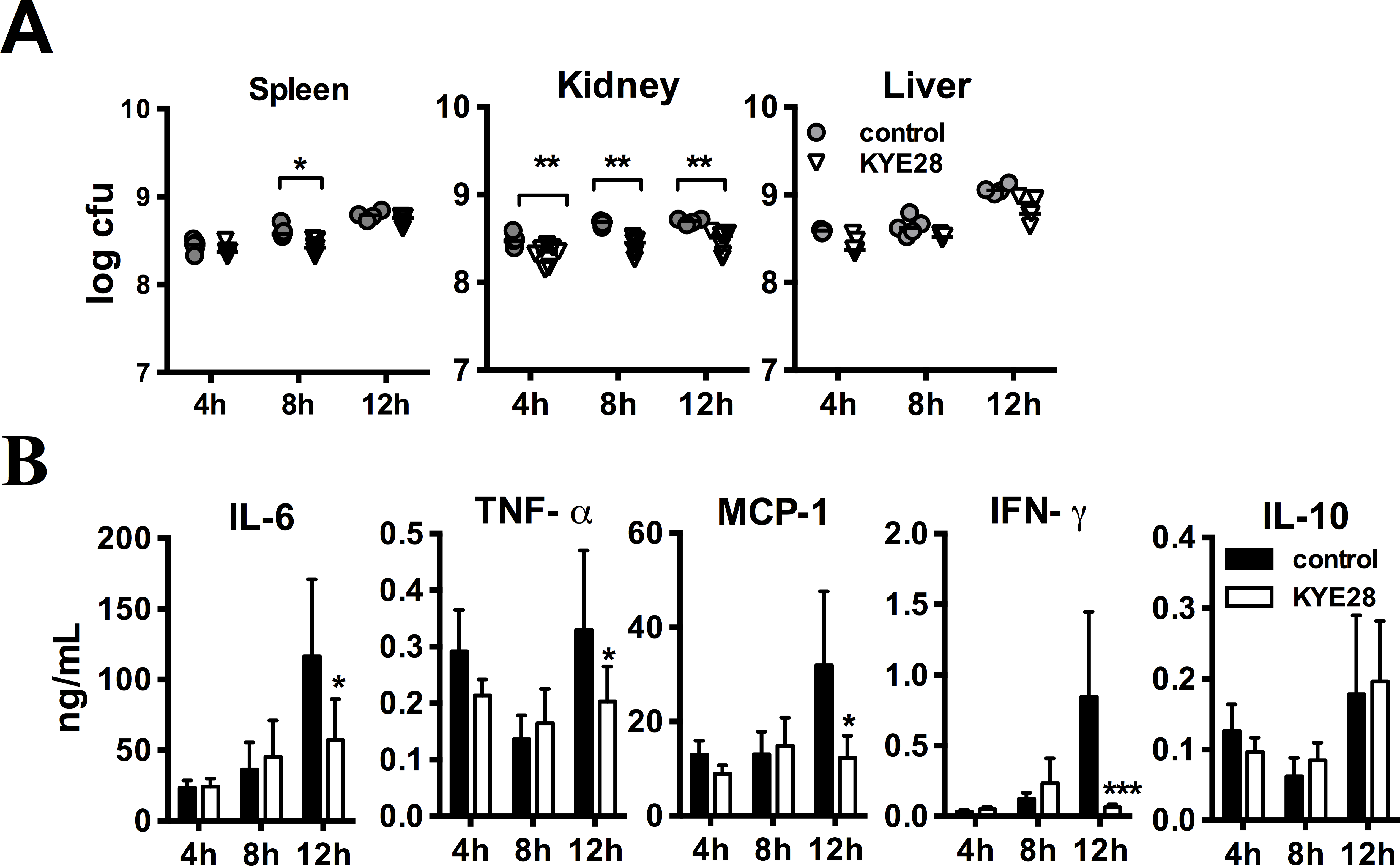

Supplement: Figure S8 — Evaluation of KYE28 treatment in a Pseudomonas infection model in vivo. (A-B) C57BL/6 mice were infected i.p. with 2×10^9^ cfu/mL P. aeruginosa 15159. KYE28 (0.5 mg) was subcutaneously injected one h after infection. (A) Bacterial counts in the indicated organs were analyzed after a time period of 4, 8, and 12 h. (Control 4 h n = 5, 8 h n = 5, 12 h n = 4; KYE28 n = 7/group). (B) In parallel, the indicated cytokines were analyzed in plasma from those mice (Control n = 9, KYE28 n = 11). (TIF) [file pone.0102577.s008.tif]

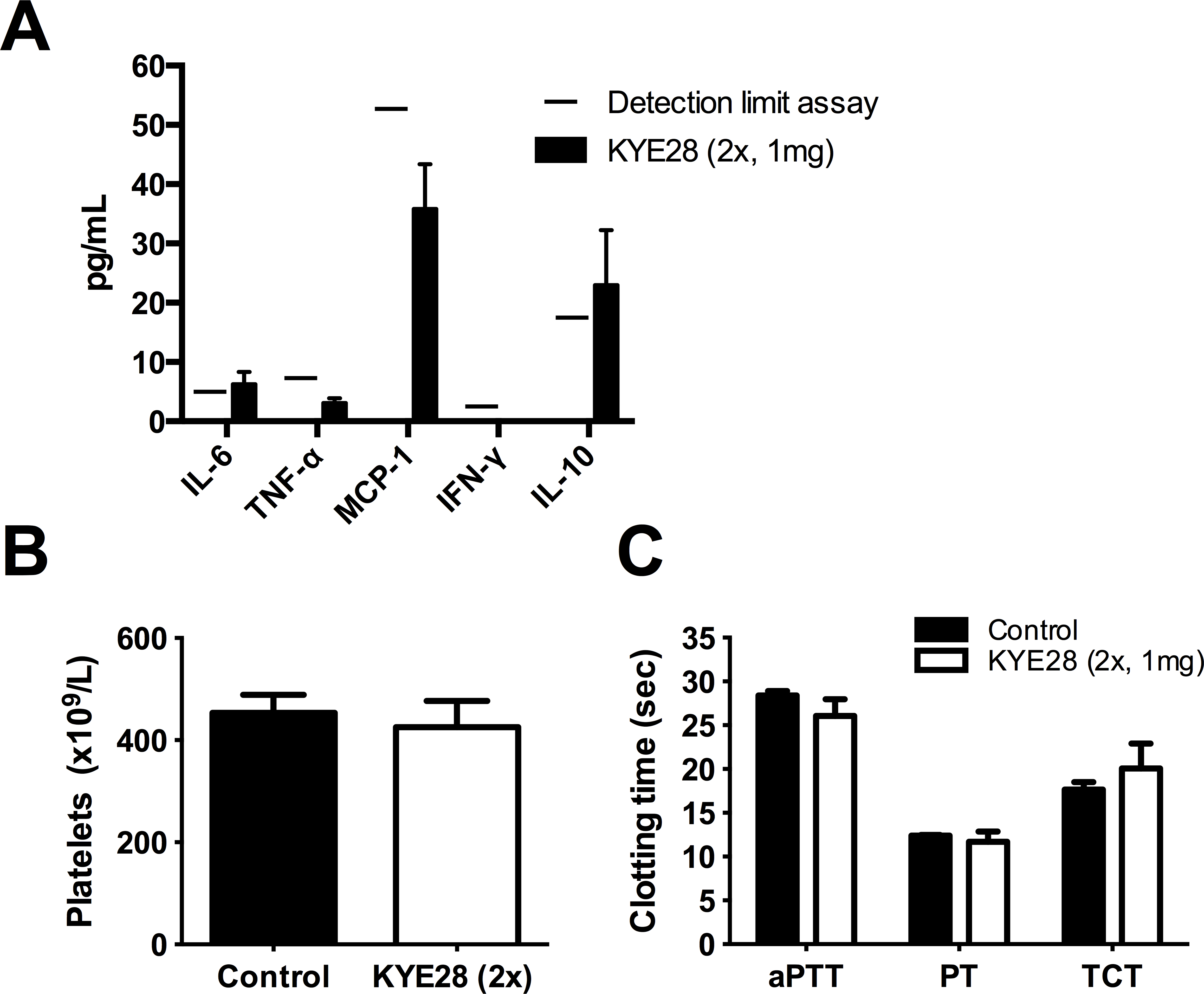

Supplement: Figure S9 — Analysis of KYE28 given alone. (A-C) Subcutaneous administration of 1 mg KYE28 or buffer (Control). Treatment was repeated 6 h post-injection and indicated parameters analyzed 12 h post-injection. (A) Cytokines determined in plasma are presented with the corresponding detection limits of the assay. (B) Determination of platelets. (C) Measurement of activated partial thromboplastin time (aPTT) and prothrombin time (PT) in mouse plasma (n = 6/group). (TIF) [file pone.0102577.s009.tif]
