# Supplementary material for: A Peptide of Heparin Cofactor II Inhibits Endotoxin-Mediated Shock and Invasive Pseudomonas aeruginosa Infection
Source: PLoS One. 2014 Jul 21;9(7):e102577. doi: 10.1371/journal.pone.0102577 (PMC4105479; doi:10.1371/journal.pone.0102577)
Supplement: Method S1 — Lactate dehydrogenase (LDH) assay. (DOCX) [file pone.0102577.s010.docx]

**Supplemental method S1**

**Lactate dehydrogenase (LDH) assay**

RAW-Blue^TM^ and THP1-XBlue^TM^-CD14 cells were stimulated as described for the NF-κB activation assay. The LDH release of the cells was quantified using the TOX-7 kit (Sigma-Aldrich). After overnight incubation 50 μL of cell supernatant was mixed with 100 µL LDH reagent mixture and incubated for 10 min at RT in the dark before absorption was measured at 490 nm. The percentage of LDH release was calculated as follows: ((A_490_ sample - A_490_ negative control)*100)/(A_490_ positive control - A_490_ negative control). The positive control consistent of cells lysed with LDH lysis buffer (containing 1% Triton X-100) for 45 min, at 37° C considered as 100 % LDH release. The negative control were cells treated with buffer only.

In another set of experiments HaCat cells (3000 cells/well), grown in serum-free keratinocyte medium supplemented with bovine pituitary extract and recombinant EGF (Invitrogen) were used to evaluate effects of KYE28 or LL-37 at the indicated concentrations either in serum-free keratinocyte medium or in keratinocyte medium supplemented with 20% human serum. The LDH release was determined as above. Results represent mean values from triplicate measurements and are given as fractional LDH release compared with the positive control.
