# Supplementary material for: A Peptide of Heparin Cofactor II Inhibits Endotoxin-Mediated Shock and Invasive Pseudomonas aeruginosa Infection
Source: PLoS One. 2014 Jul 21;9(7):e102577. doi: 10.1371/journal.pone.0102577 (PMC4105479; doi:10.1371/journal.pone.0102577)
Supplement: Method S2 — Cell viability assay (MTT assay). (DOCX) [file pone.0102577.s011.docx]

**Supplemental method S2**

**Cell viability assay (MTT assay)**

HaCaT cells (3,000 cells/well) were grown in serum-free keratinocyte medium, containing bovine pituitary extract and recombinant EGF to confluence. KYE28 and LL-37 were then added at the indicated concentrations. In some experiments the effect of KYE28 or LL-37 (60 μM) on cell viability in medium containing 20% human serum was tested. After overnight incubation, 20 μL of a sterile filtered MTT [3-(4,5-dimethylthiazolyl)-2,5-diphenyl-tetrazolium bromide; Sigma-Aldrich] solution (5 mg/mL in PBS) were added to each well and cells were further incubated for 1 h in 5 % CO_2_ at 37°C allowing them to form the blue formazan product. After removal of the cell supernatant 100 μL of 100% dimethyl sulfoxide (DMSO) were added and plates were then gently swirled for 10 min at room temperature to dissolve the precipitate. The absorbance was monitored at 550 nm, and the results given represent mean±SEM. Positive control samples contained only medium, whereas for negative controls samples, cells were lysed with lysis buffer containing 1% Triton X-100.
