# Supplementary material for: A Peptide of Heparin Cofactor II Inhibits Endotoxin-Mediated Shock and Invasive Pseudomonas aeruginosa Infection
Source: PLoS One. 2014 Jul 21;9(7):e102577. doi: 10.1371/journal.pone.0102577 (PMC4105479; doi:10.1371/journal.pone.0102577)
Supplement: Method S3 — Hemolysis assay. (DOCX) [file pone.0102577.s012.docx]

**Supplemental method S3**

**Hemolysis assay**

Citrate-blood was diluted (1:1) with phosphate-buffered saline (PBS). The cells were then incubated for 1 h at 37°C (end-over-end rotation) in the presence of KYE28 or LL-37 (60 μM). Triton X-100 (2%; Sigma-Aldrich, St. Louis) was used to induce 100 % lysis in positive control samples. After the incubation, samples were centrifuged at 900 **x** g for 10 min. and the supernatants were transferred to a 96-well microtiter plate. The hemoglobin release was measured by determining the absorbance of the samples at 550 nm and is presented as the percentage of Triton X-100-induced hemolysis.
