# Supplementary material for: A Peptide of Heparin Cofactor II Inhibits Endotoxin-Mediated Shock and Invasive Pseudomonas aeruginosa Infection
Source: PLoS One. 2014 Jul 21;9(7):e102577. doi: 10.1371/journal.pone.0102577 (PMC4105479; doi:10.1371/journal.pone.0102577)
Supplement: Method S4 — Coagulation assay. (DOCX) [file pone.0102577.s013.docx]

**Supplemental method S4**

**Coagulation assay**

For determination of prothrombin time (PT), as well as thrombin clotting time (TCT), a thromboplastin reagent (Trinity Biotech) and Thrombin reagent (Technoclone) were used, respectively. Hundred μL of fresh citrate plasma, together with 20 μM KYE28 or buffer were pre-warmed for 60 sec at 37°C before clot formation was initiated by adding 100 μL of clotting reagent. To determine the activated partial thromboplastin time (aPTT), 100 μL of Dapptin, a kaolin-containing solution (Technoclone) were added to the plasma-peptide mix and incubated for 200 sec before clot formation was initiated by adding 100 μL of 30 mM fresh CaCl_2_ solution. All clotting times were analyzed using a coagulometer (Amelung, Lemgo, Germany).
